# Abstracts from the 1st JoPPP Conference on Pharmaceutical Policy and Practice: Shah Alam, Malaysia. 28-29 August 2019

**DOI:** 10.1186/s40545-019-0201-2

**Published:** 2020-01-29

**Authors:** Salman Mehmood, Syed Muhammad Farid Hasan, Chinara Maratovna Razzakova, Liliya Eugenevna Ziganshina, Azjargal Ganbat, Tsetsegmaa Sanjjav, Bruce Sunderland, Gantuya Dorj, Gereltuya Dorj, Satibi Satibi, M. Rifqi Rokhman, Hardika Aditama, Ika Kartini, Rini Ambarsari, Fajar Pramesti, Peng Yeow Loh, Siew Siang Chua, Mahmathi Karuppannan, Rohit Kumar Verma, Thomas Paraidathathu, Nur Akmar Taha, Wei Wen Chong, M. Rozaini Rosli, Chin Fen Neoh, Mahmathi Karuppannan, W. Nazariah W. Hassan, Mahani Mahmud, Afifah Rahimi, David Bin-Chia Wu, Ren Ee Teh, Adliah Mhd Ali, Mohammed Mustapha, Hadzliana Zainal, Balamurugan Tangiisuran, Sabariah Noor Harun, Irene Looi, Norsima Nazifah Sidek, Khairul Azmi Ibrahim, Loo Keat Wei, Lee Keng Yee, Zariah Abdul Aziz, Khalid A. Al-Sunaidar, Noorizan Abd Aziz, Yahaya Hassan, Mohamed Abdul Hameed, Nur Fatihah Binti Shaari, Mahmathi Karuppannan, Hasnah Ismail, Yuet Yen Wong, Chin Fen Neoh, Rifqa Danisha Nadhrah Ramlan, Wan Asma Najiha A. Raman, Syazani Salman Radzaini, Norazila Abdul Ghani, Qi Ying Lean, Chin Fen Neoh, Yuet Yen Wong, A. Md Shariff, M. Karuppannan, S. Gnanasan, A. Aziz N, Muhammad Helmi Zaini, Mohd Shahezwan Abd Wahab, Dulmaa Lkhagvasuren, Enkhjargal Dorjbal, Qurratul-ain Leghari, Muhammad Shahzad Aslam, Neelum Malick, Sadia Kashif, Sharmeen Bawani, Nur Sufiza Bt Ahmad, Ernieda Hatah, Mohd Makmor-Bakry, Raveena Amee Nagaria, Syed Shahzad Hasan, Zaheer Ud-Din Babar, Erwin Martinez Faller, Jesa Madelo, Edward Agravante Tolentino, Zakiah Mohd Noordin, Mahmathi Karuppannan, Neoh Chin Fen, Nor Haizan Ibrahim Ghazali, F. El-Dahiyat, M. Rashrash, S. Abuhamdah, R. Abu Farha, Amjad Khan, Amer Hayat Khan, Syed Azhar Syed Sulaiman, Azreen Syazril Adnan, Saima Mushtaq, Maisarah Mohamad Fadzil, Muhammad Hadif Syahmi Mohd Akmal, Yuet Yen Wong, Chin Fen Neoh, Qi Ying Lean, Christina Malini, Noor Azizah Binti Abdul Wahab, Ahmad Fuad Shamsuddin, Ahmad Fauzi Dali, Norfarahin Abd Patah, Noorizam Ibrahim, Min H. Cheah, Eon T. Gan, Nur J. Azman, Najma Kori, Petrick Periyasamy, Siti-Azdiah Abdul-Aziz, Wan Ismahanisa Ismail, Mohamed Azmi Ahmad Hassali, Maryam Farooqui, Muhammad Nabil Fikri Roslan, Nurhidayah Ab Rahim, Syarifah Masyitah Habib Dzulkarnain, Audrey K. Chigome, Moliehi Matlala, Johanna C. Meyer, Nur Hazirah Izzati Zaidi, Kamaliah Md Saman, Mathumalar Loganathan Fahrni, Fajaratunur A. Sani, Shubashini Gnanasan, Mahmathi Karuppannan, Dema Nasser Alhebs, Nur Sabiha Md Hussin, Shubashini Gnanasan, Mahmathi Karupannan, Yogheswaran Gopalan, Badamkhand Gankhulug, Otgonbileg Tegshee, Gereltuya Dorj, Tsetsegmaa Sanjjav, Bruce Sunderland, Gantuya Dorj, Azjargal Ganbat, Gereltuya Dorj, Tsetsegmaa Sanjjav, Bruce Sunderland, Gantuya Dorj, D. Ariunaa, S. Purevsuren, S. Tugsbileg, B. Boditsetseg, D. Baigalmaa, B. Bolor, B. Otgonbat, P. Mandahnaran, Asma Alhatlani, Nujud Alshehaitan, Amjad Alherabi, Maryam Farooqui, M. S. Gill, A. H. Basari, M. A. Adnan, M. A. Mat Rahim, Nurul Atika Mohd Fauzi, Nurin Hasya Hamidi, Muhammad Amin Azizul Rahman, Norazila Abdul Ghani, Qi Ying Lean, Chin Fen Neoh, Yuet Yen Wong, Izzah Zahra Nasrudin, Nur Izzati Mohd Zaki, Muhammad Fareez Nazwaad Ismail, Norazila Abdul Ghani, Qi Ying Lean, Chin Fen Neoh, Yuet Yen Wong, Adeel Aslam, Che Suraya Zin, Norny Syafinaz, Syed Imran Ahmad, Shazia Jamshed, H. Yahaya, H. A. Suriana, A. W. Izyan, R. Amlizan, Tuangrat Phodha, Arthorn Riewpaiboon, Kumthorn Malathum, Peter C. Coyte, Nursyuhadah Othman, Yuet Yen Wong, Qi Ying Lean, Nurain Mohd Noor, Chin Fen Neoh, Sunee Lertsinudom, Pawalee Niamtaworn, Sirirat Tanpichart, Doaa Alkhalidi, Shazia Qasim Jamshed, Ramadan Mohamed Elkalmi, Mirza Rafi Baig, Adeel Aslam, Mohamed Azmi Hassali, Ezlina Usir, Nurul Hafizah Azman, Hasnah Ismail, Mathumalar Loganathan Fahrni, Noor Razilah Abdul Rafar, Chin Fen Neoh, Muhamad Faiz Othman, Nur Zuhaida Zainal Bahrin, Wan Sazrina Wan Zaid, Fauziah Zamri, Nurita Andayani, Farida Ariani, Prih Sarnianto, Zarith Sofia, Faiza Naimat, Mathumalar Loganathan Fahrni, Beh Huai Min, Nor Liana Che Yaacob, Irma Wati Ngadimon, Mathumalar Loganathan Fahrni, Safwanah Rahman, Nasrul Nazim Husna Zunaidi, Azyyati Mohd Suhaimi, M. M. Manan, N. L. Jamaluddin, M. M. Manan, A. L. Nadiah Loke, Muhammad Anwar Nawab Khan, Nurin Alya Bt Saroge, Muhammad Anwar Nawab Khan, Wang S. Ng, Pey C. Wong, Siti N. Md Said

**Affiliations:** 1 Macter International Limited, F-216, S.I.T.E, Karachi, Pakistan; 2 Department of Pharmaceutics, Faculty of Pharmacy & Pharmaceutical Sciences University of Karachi, Karachi, Pakistan; 3 Research & Education Centre for Evidence-Based Medicine Cochrane Russia, Kazan Federal University, Kazan, Republic of Tatarstan Russian Federation; 4 School of Pharmacy, Mongolian National University of Medical Sciences, S. Zorig Street, Ulaanbaatar, 14210 Mongolia; 5 Mongolian University of Pharmaceutical Sciences, Sonsgolon Road-5, Songinokhairkhan District, Ulaanbaatar, 18130 Mongolia; 6 Drug Research Institute, Monos group, Sonsgolon Road-5, Songinokhairkhan District, Ulaanbaatar, 18130 Mongolia; 7 Faculty of Health Sciences, Curtin University, Kent Street, Bentley, Perth, Western Australia 6102 Australia; 8 School of Public Health, Mongolian National University of Medical Sciences, S. Zorig Street, Ulaanbaatar, 14210 Mongolia; 9 Departemen Farmasetika, Faculty of Pharmacy Universitas Gadjah Mada, Yogyakarta, Indonesia; 10 School of Pharmacy, Taylor’s University, Subang, Selangor Malaysia; 11 Faculty of Pharmacy, Universiti Technologi MARA, Shah Alam, Malaysia; 12 Department of Pharmacy Practice, School of Pharmacy, International Medical University, 57000 Kuala Lumpur, Malaysia; 13 Faculty of Health and Medical Sciences Taylor’s University, 47500 Subang Jaya, Malaysia; 14 Faculty of Pharmacy, Cyberjaya University College of Medical Sciences, 63000 Cyberjaya, Selangor Malaysia; 15 Faculty of Pharmacy, Universiti Kebangsaan Malaysia, 50300 Kuala Lumpur, Malaysia; 16 Faculty of Pharmacy, Level 11, FF1 Building, UiTM Puncak Alam Campus, ., Selangor Malaysia; 17 Bandar Pasir Mas Health Clinic, Jalan Hospital, Pasir Mas, Kelantan Malaysia; 18 School of Pharmacy, Monash University Malaysia, Bandar Sunway, Selangor Malaysia; 19 Asian Centre for Evidence Synthesis in Population, Implementation and Clinical Outcomes, Health and Well-Being Cluster, Global Asia in the 21st Century Platform, Monash University Malaysia, Bandar Sunway, Selangor Malaysia; 20 Faculty of Pharmacy, Universiti Kebangsaan Malaysia, 50300 Kuala Lumpur, Malaysia; 21 School of Pharmaceutical Sciences, Universiti Sains Malaysia, 11800 USM, Gelugor, Pulau Pinang Malaysia; 22 Department of Clinical Pharmacy and Pharmacy Practice, Ahmadu Bello University Zaria, Zaria, Nigeria; 23 National Poison Centre, Universiti Sains Malaysia, 11800 USM, Gelugor, Pulau Pinang Malaysia; 24 Clinical Research Centre, Hospital Seberang Jaya, Perai, Pulau Pinang Malaysia; 25 Clinical Research Centre, Hospital Sultanah Nur Zahirah, Kuala Terengganu, Terengganu Malaysia; 26 Department of Biological Science, Faculty of Science, Universiti Tunku Abdul Rahman, Kampar Campus, Kampar, Perak Malaysia; 27 National Clinical Research Centre, Kuala Lumpur, Malaysia; 28 Clinical Research Centre, Hospital Sultanah Nur Zahirah, Kuala Terengganu, Terengganu Malaysia; 29 Department of Pharmacy Practice, Faculty of Pharmacy, Universiti Teknologi MARA (UiTM), Puncak Alam, Selangor Malaysia; 30 Department of Pharmacy Practice, School of Pharmacy, Management and Science University, Shah Alam, Selangor Malaysia; 31 Faculty of Pharmacy, Universiti Teknologi MARA (UiTM), Selangor Branch, Puncak Alam Campus, 42300 Puncak Alam, Selangor Malaysia; 32 Faculty of Pharmacy, Universiti Teknologi MARA, Penang Branch, Bertam Campus, 12300 Kepala Batas, Penang Malaysia; 33 Faculty of Pharmacy, Universiti Teknologi MARA, Cawangan Pulau Pinang, Kampus Bertam, Penang, Malaysia; 34 Pharmacy Department, Hospital Sultanah Bahiyah, Alor Setar, Malaysia; 35 Vector-Borne Diseases Research Group (VERDI), Pharmaceutical and Life Sciences CoRe, Universiti Teknologi MARA, Shah Alam, Selangor Malaysia; 36 Faculty of Pharmacy, Universiti Teknologi MARA, Cawangan Selangor, Kampus Puncak Alam, Kuala Selangor, Selangor Malaysia; 37 Collaborative Drug Discovery Research (CDDR) Group, Pharmaceutical and Life Sciences Community of Research, Universiti Teknologi MARA, Shah Alam, Selangor Malaysia; 38 Department of Pharmacy, Hospital Tengku Ampuan Rahimah, Klang, Selangor Malaysia; 39 Department of Pharmacy Practice, Universiti Teknologi MARA, Puncak Alam Campus, Shah Alam, Selangor Malaysia; 40 Department of Pharmacy Practice, Malaysian Science University (MSU), Shah Alam Campus, Shah Alam, Selangor Malaysia; 41 Department of Pharmacy Practice, Faculty of Pharmacy, Universiti Teknologi MARA (UiTM), 42300 Bandar Puncak Alam, Selangor Malaysia; 42 School of Pharmacy, Mongolian National University of Medical Sciences, Ulaanbaatar, Mongolia; 43 Department of Pharmaceutical Chemistry, Faculty of Pharmacy, Ziauddin University, Clifton Karachi, Pakistan; 44 Department of Pharmaceutical Chemistry, Faculty of Pharmacy, University of Karachi, Karachi, Pakistan; 45 Department of Chemistry, Xiamen University Malaysia, 43900 Sepang, Malaysia; 46 Department of Pharmacology, Faculty of Pharmacy, Ziauddin University, Clifton Karachi, Pakistan; 47 Department of Pharmaceutics, Faculty of Pharmacy, University of Karachi, Karachi, Pakistan; 48 Discipline of pharmacy practice, Faculty of Pharmacy, Universiti Kebangsaan Malaysia, Jalan Raja Muda Abd Aziz, 50300 Kuala Lumpur, Malaysia; 49 Pharmaceutical Services Division, Ministry of Health Malaysia, 46350 Petaling Jaya, Selangor Malaysia; 50 Department of Pharmacy, University of Huddersfield, Queensgate, Huddersfield, HD1 3DH UK; 51 Research Management Center, Tagum Doctors College, Inc., Visayan Village, 8100 Tagum, Philippines; 52 Pharmacy Department, Tagum Doctors College, Inc., Visayan Village, 8100 Tagum, Philippines; 53 Supply Chain and Business Development, Regicon Healthcare Inc., Makati City, Philippines; 54 Department of Pharmacy Practice, Faculty of Pharmacy, Universiti Teknologi MARA, Bandar Puncak Alam, Selangor Malaysia; 55 Pharmacy Department, Klinik Kesihatan Kuala Lumpur, Jalan Temerloh, Taman Tasik Titiwangsa, 53200 Kuala Lumpuur, Malaysia; 56 College of Pharmacy, Al-Ain University of Science and Technology, Al Ain Campus, Al Ain, United Arab Emirates; 57 College of Pharmacy, Al-Ain University of Science and Technology, Abu Dhabi Campus, Abu Dhabi, United Arab Emirates; 58 Department of Biopharmaceutics and Clinical Pharmacy, Faculty of Pharmacy, The University of Jordan, Amman, Jordan; 59 Department of Clinical Pharmacy and Therapeutics, Faculty of Pharmacy, Applied Science Private University, Amman, Jordan; 60 Discipline of Clinical Pharmacy, School of Pharmaceutical Sciences, Universiti Sains Malaysia, 11800 Penang, Malaysia; 61 Chronic Kidney Disease Resource Centre, School of Medical Sciences, Health Campus, Universiti Sains Malaysia, 16150 Kubang Kerain, Kelantan Malaysia; 62 Department of Pharmacy, Quaid-i-Azam University, Islamabad, 45320 Pakistan; 63 Health Care Biotechnology Department, Atta ur Rahman School of Applied Biosciences, National University of Science & Technology, Islamabad, 44000 Pakistan; 64 Faculty of Pharmacy, Universiti Teknologi MARA, Cawangan Pulau Pinang, Kampus Bertam, Penang, Malaysia; 65 Faculty of Pharmacy, Universiti Teknologi MARA, Cawangan Selangor, Kampus Puncak Alam, Kuala Selangor, Selangor Malaysia; 66 Collaborative Drug Discovery Research (CDDR) Group, Pharmaceutical and Life Sciences Community of Research, Universiti Teknologi MARA, Shah Alam, Selangor Malaysia; 67 Vector-Borne Diseases Research Group (VERDI), Pharmaceutical and Life Sciences CoRe, Universiti Teknologi MARA, Shah Alam, Selangor Malaysia; 68 Hospital Seberang Jaya, Penang, Pulau Pinang Malaysia; 69 School of Pharmaceutical Sciences, Royal College of Medicine, Penang, Perak Malaysia; 70 Department of Pharmacy Practice, Faculty of Pharmacy, Universiti Teknologi MARA, 42300 Bandar Puncak Alam, Selangor Malaysia; 71 Faculty of Pharmacy, National University of Malaysia (UKM), Bangi, Malaysia; 72 Infectious Disease Clinic, Hospital Sultan Haji Ahmad Shah, Temerloh Pahang, Malaysia; 73 Pharmacy Department, Hospital Canselor Tuanku Muhriz, Kuala Lumpur, Malaysia; 74 Infectious Disease Clinic, Hospital Canselor Tuanku Muhriz, Kuala Lumpur, Malaysia; 75 Faculty of Health Sciences UniversitiTeknologi MARA, Penang, Malaysia; 76 School of Pharmaceutical Sciences, UniversitiSains Malaysia, Penang, Malaysia; 77 Department of Pharmacy Practice, Unaizah College of Pharmacy, Qassim University, Qassim, Saudi Arabia; 78 Department of Public Health Pharmacy and Management, School of Pharmacy, Sefako Makgatho Health Sciences University, Ga-Rankuwa, South Africa; 79 Department of Pharmacy Practice, Faculty of Pharmacy, Universiti Teknologi MARA Cawangan Selangor Kampus Puncak Alam, 42300 Bandar Puncak Alam, Selangor Malaysia; 80 Muar District Health Clinic, Muar, Johor Malaysia; 81 Faculty of Pharmacy, Department Of Pharmacy Practice, University Technology MARA (UiTM), Shah Alam, Malaysia; 82 Unizah College of Pharmacy, Qassim University, Unayzah, Saudi Arabia; 83 Department of Pharmacy Practice, University Teknologi MARA, Bandar Puncak Alam, Selangor Malaysia; 84 School of Pharmacy, Mongolian National University of Medical Sciences, S. Zorig Street, Ulaanbaatar, 14210 Mongolia; 85 The Third Central Hospital of Mongolia, Bayangol District, 10th khoroolol-2, Ard Ayush Avenue, Ulaanbaatar, Mongolia; 86 Drug Research Institute, Monos group, Sonsgolon Road-5, Songinokhairkhan District, Ulaanbaatar, 18130 Mongolia; 87 Faculty of Health Sciences, Curtin University, Kent Street, Bentley, Perth, Western Australia 6102 Australia; 88 School of Public Health, Mongolian National University of Medical Sciences, S. Zorig Street, Ulaanbaatar, 14210 Mongolia; 89 School of Pharmacy, Mongolian National University of Medical Sciences, S. Zorig Street, Ulaanbaatar, 14210 Mongolia; 90 Mongolian University of Pharmaceutical Sciences, Sonsgolon Road-5, Songinokhairkhan District, Ulaanbaatar, 18130 Mongolia; 91 Drug Research Institute, Monos Group, Sonsgolon Road-5, Songinokhairkhan District, Ulaanbaatar, 18130 Mongolia; 92 Faculty of Health Sciences, Curtin University, Kent Street, Bentley, Perth, Western Australia 6102 Australia; 93 School of Public Health, Mongolian National University of Medical Sciences, S. Zorig Street, Ulaanbaatar, 14210 Mongolia; 94 Department of Pharmacy Technician, School of Pharmacy, Mongolian National University of Medical Sciences, Sukhbaatar District, S. Zorig Street, Ulaanbaatar City, 14210 Mongolia; 95 Uniazah College of Pharmacy, Qassim University, Buraydah, Saudi Arabia; 96 Department of Pharmacy Practice, Unaizah College of Pharmacy , Qassim University, Buraydah, Saudi Arabia; 97 Department of Pharmacy, 95 Armed Forces Hospital, Ministry of Defense, Kuala Lumpur, Malaysia; 98 Health Services Division, MAF HQ, Ministry of Defense, Kuala Lumpur, Malaysia; 99 93 Armed Forces Medical & Dental Depot, Ministry of Defense, Kuala Lumpur, Malaysia; 100 Faculty of Pharmacy, Universiti Teknologi MARA, Cawangan Pulau Pinang, Kampus Bertam, Penang, Malaysia; 101 Pharmacy Department, Hospital Sultanah Bahiyah, Alor Setar, Malaysia; 102 Vector-Borne Diseases Research Group (VERDI), Pharmaceutical and Life Sciences CoRe, Universiti Teknologi MARA, Shah Alam, Selangor Malaysia; 103 Faculty of Pharmacy, Universiti Teknologi MARA, Cawangan Selangor, Kampus Puncak Alam, ., Selangor Malaysia; 104 Collaborative Drug Discovery Research (CDDR) Group, Pharmaceutical and Life Sciences Community of Research, Universiti Teknologi MARA, Shah Alam, Selangor Malaysia; 105 Faculty of Pharmacy, Universiti Teknologi MARA, Cawangan Pulau Pinang, Kampus Bertam, Penang, Malaysia; 106 Pharmacy Department, Hospital Sultanah Bahiyah, Alor Setar, Malaysia; 107 Vector-Borne Diseases Research Group (VERDI), Pharmaceutical and Life Sciences CoRe, Universiti Teknologi MARA, Shah Alam, Selangor Malaysia; 108 Faculty of Pharmacy, Universiti Teknologi MARA, Cawangan Selangor, Kampus Puncak Alam, ., Selangor Malaysia; 109 Collaborative Drug Discovery Research (CDDR) Group, Pharmaceutical and Life Sciences Community of Research, Universiti Teknologi MARA, Shah Alam, Selangor Malaysia; 110 Pharmacy Practice Department, Kulliyyah of Pharmacy, International Islamic University Malaysia, Kuantan, Pahang Malaysia; 111 Pharmacy Practice Department, School of Pharmacy, International Medical University, Kuala Lumpur, Malaysia; 112 Management and Science University, Shah Alam, Malaysia; 113 Pharmacy Enforcement Division, Ministry of Health Malaysia, Putrajaya, Malaysia; 114 Cyberjaya University College of Medical Sciences, Cyberjaya, Malaysia; 115 MARA University of Technology, Kuala Lumpur, Malaysia; 116 Center of Excellence in Pharmacy Practice and Management Research, Drug Information and Consumer Protection Center, Faculty of Pharmacy, Thammasat University, Bangkok, Pathumthani Thailand; 117 Faculty of Pharmacy, Mahidol University (Graduate Program), Bangkok, Thailand; 118 Division of Infectious Disease, Department of Medicine, Faculty of Medicine Ramathibodi Hospital, Mahidol University, Bangkok, Thailand; 119 Institute of Health Policy Management and Evaluation, School of Public Health University of Toronto, Toronto, Ontario Canada; 120 Faculty of Pharmacy, Universiti Teknologi MARA (UiTM), Cawangan Selangor, Kampus Puncak Alam, 42300 Bandar Puncak Alam, Selangor Darul Ehsan Malaysia; 121 Faculty of Pharmacy, Universiti Teknologi MARA (UiTM), Cawangan Pulau Pinang, Kampus Bertam, 13200 Kepala Batas, Pulau Pinang Malaysia; 122 Vector-Borne Diseases Research Group (VERDI), Pharmaceutical and Life Sciences CoRe, Universiti Teknologi MARA, Shah Alam, Selangor Malaysia; 123 Endocrine Unit, Hospital Putrajaya, Precint 7, 62250 Putrajaya, Malaysia; 124 Collaborative Drug Discovery Research (CDDR) Group, Pharmaceutical and Life Sciences Community of Research, Universiti Teknologi MARA (UiTM), 40450 Shah Alam, Selangor Darul Ehsan Malaysia; 125 Clinical Research Centre, Hospital Putrajaya, Precint 7, 62250 Putrajaya, Malaysia; 126 Division of Clinical Pharmacy, Faculty of Pharmaceutical Sciences, Khon Kaen University, London, UK; 127 Division of Clinical Pharmacy, Faculty of Pharmacy, Thammasat University, London, UK; 128 Community Pharmacy Association (Thailand), Bangkok, Thailand; 129 Department of Clinical Pharmacy & Pharmacotherapeutics, Dubai Pharmacy College, Dubai, United Arab Emirates; 130 Department of Pharmacy Practice, Kulliyyah of Pharmacy, IIUM, 25200 Kuantan, Pahang Malaysia; 131 Department of Pharmacy Practice, Faculty of Pharmacy, Universiti Teknologi Mara, Shah Alam, Malaysia; 132 Department of Clinical Pharmacy & Pharmacotherapeutics, Dubai Pharmacy College, Dubai, United Arab Emirates; 133 Discipline of Social and Administrative Pharmacy, School of Pharmaceutical Sciences, Universiti Sains Malaysia, 11800 Penang, Malaysia; 134 Department of Pharmacy Practice, Faculty of Pharmacy, Universiti Teknologi MARA (UiTM) Cawangan Selangor, Kampus Puncak Alam, 42300 ., Selangor Malaysia; 135 Faculty of Pharmacy, Universiti Teknologi MARA, Puncak Alam, Selangor Malaysia; 136 Klinik Kesihatan Senawang, Ministry of Health, Seremban, Malaysia; 137 Faculty of Pharmacy, Cyberjaya University College of Medical Sciences Malaysia, Selangor, Malaysia; 138 Faculty of Pharmacy, Pancasila University, Jakarta, Indonesia; 139 School of Pharmacy, Management & Science University, 40100 Shah Alam, Malaysia; 140 Universiti Teknologi MARA Cawangan Selangor Kampus Puncak Alam, Selangor, Malaysia; 141 Unit of Pharmacy Practice, School of Pharmacy, Management and Science University, University Drive, Off Persiaran Olahraga, 40100 Shah Alam, Selangor Malaysia; 142 Department of Pharmacy Practice, Faculty of Pharmacy, Universiti Teknologi MARA Cawangan Selangor Kampus Puncak Alam, 42300 Bandar Puncak Alam, Selangor Malaysia; 143 Department of Pharmacy Practice, Faculty of Pharmacy, Mahsa University, Jln SP 2, Bandar Saujana Putra, 42610 Jenjarom, Selangor Malaysia; 144 Faculty of Pharmacy, Universiti Teknologi MARA (UiTM) Selangor, Kampus Puncak Aam, 42300 Bandar Puncak Alam, Selangor Malaysia; 145 Department of Pharmacy Practice, Faculty of Pharmacy, Universiti Teknologi MARA (UiTM) Selangor, Kampus Puncak Aam, 42300 Bandar Puncak Alam, Selangor Malaysia; 146 Faculty of Pharmacy, UiTM, Puncak Alam Campus, Puncak Alam, Malaysia; 147 School of Pharmacy, KPJ Healthcare University College, Nilai, Malaysia; 148 Faculty of Pharmacy, UiTM, Puncak Alam Campus, Puncak Alam, Malaysia; 149 School of Pharmacy, KPJ Healthcare University College, Nilai, Malaysia; 150 Department of Pharmacy Practice, Faculty of pharmacy, Universiti Teknologi MARA (UiTM), 42300 Bandar Puncak Alam, Selangor Malaysia; 151 Department of Pharmacy practice, Faculty of Pharmacy, Universiti Teknologi MARA (UiTM), 42300 Bandar Puncak Alam, Selangor Malaysia; 152 Faculty of Pharmacy, Universiti Teknologi MARA (UiTM) Selangor, Kampus Puncak Aam, 42300 Bandar Puncak Alam, Selangor Malaysia; 153 Department of Pharmacy, Sultanah Aminah Hospital, Johor Bahru, Malaysia; 154 Faculty of Pharmacy, Universiti Institute Technology MARA, Puncak Alam, Malaysia

## 01 Data Integrity and the role of the Pharmaceutical Industry

### Salman Mehmood^1^, Syed Muhammad Farid Hasan^2^

#### ^1^Assistant Manager Quality Assurance,Macter International Limited,F-216, S.I.T.E., Karachi, Pakistan; ^2^Associate Professor & Chairman,Department of Pharmaceutics,Faculty of Pharmacy & Pharmaceutical Sciences,University of Karachi,Karachi,Pakistan.

##### **Correspondence:** Salman Mehmood (salman_731a@hotmail.com)

Pharmaceutical industry is one of the main contributors of public health. In a pharmaceutical industry, Data integrity is one of the most important aspect that relates to drug quality, safety, efficacy and purity. Recently, many industries have been given warning letters due to the violation of data integrity and strict action had been taken by international and local regulatory authorities. Data integrity should be a part of pharmaceutical policy. It claims that product has been manufactured after meeting the predetermined specification and quality attributes and showed compliance of testing methods according to the guidelines given in official books. The FDA, USA considers integrity of data, from initial step where it is generated, and extending throughout its life cycle, to be a critical component which ensures that only high quality and safe drugs are manufactured. Hence, it is essential to record each and every detail of drug manufacturing and testing which should comply with cGMP practices. In future, the completeness, consistency, and accuracy of the data will decide the fate of the pharmaceutical companies.

## 02 Essential Medicines Prices in Russian Public Health Facilities in 2010 and 2015

### Chinara Maratovna Razzakova, Liliya Eugenevna Ziganshina

#### Research & Education Centre for Evidence-Based Medicine Cochrane Russia, Kazan Federal University, Kazan, Republic of Tatarstan, Russian Federation

##### **Correspondence:** Chinara Maratovna Razzakova (razzakova345@gmail.com)

Effective medicine procurement is key to improve rational use of financial resources, and to improve Universal Health Coverage. Over the last decades drug prices in Russia have been rising. In 2013 the Government decree on the rules for medicinal products procurement came into force as a high level intervention to reduce medicine prices. Health Action International (HAI), in collaboration with the World Health Organization (WHO), has developed methodology to measure medicine price comparisons, medicine availability and affordability, changes over time and cross-country comparisons [1]. The objective of our study was to assess effects of governmental interventions aimed at drug price reductions by comparing medicine prices in public hospitals in Russia in 2010 and 2015.We used WHO/HAI methodology to collect and process collected data [1]. We obtained procurement prices from state owned pharmacies of public hospitals of the Republic of Tatarstan, Russian Federation (medicines for hospitals). We surveyed 95 medicines totally: 30 medicines - from the WHO/HAI recommended list (14 global list and 16 regional list) and 65 - cardiovascular medicines (supplementary list). For each medicine, we collected prices for the lowest priced generic (LPG) and the original brand (OB). We calculated median price ratios (MPR) for each listed product. The MPR was the ratio of the local median unit price of a given medicine to the median unit price of the international reference prices (IRP) obtained from the Management Sciences for Health Price Indicator Guide for the years 2010 and 2015 [2]. MPR value of 1 or less indicates that the collected prices are within acceptable range and not excessive. Not all medicines selected for the study were purchased by the surveyed public hospitals. We collected data on state procurement of 61 medicines in 2010 and 29 medicines in 2015.In 2010, 48 medicines were procured as LPGs (11 – global list, 12 – regional list and 25 – supplementary list) and 13 – as OBs (2 – global list, 2 – regional list and 9 – supplementary list). The prices of medicines exceeded the IRP by 3 to 12 times, with the MPR for LPGs being 2.9 [1,8–5,5] and for OBs – 12,3 [4,4–14,9]. In 2015 public hospitals procured 28 medicines as generic versions and only 1 medicine as an originator brand (amiodarone 50 mg/ml). These 28 LPGs consisted of 6 medicines from the global list, 3 medicines – regional list and 19 – supplementary list. This was due to the 2013 Decree of the Government of the Russian Federation, which restricted procurement of originator brands, by means of creating a special list of medicines allowed for procurement as originator brands [3]. In 2015, the MPR for LPGs was 1.1 [0.2–2.6] and for OB – 2.0. We see the marked decrease of state procurement medicine prices in 2015 compared to 2010, by at least 2 times for LPMs, and 6 times for OBs. Interventions at the highest governmental level, aimed at lowering medicine prices, proved successful in terms of the reduction of public procurement of originator brands (OBs) and preferential procurement of lowest priced generics (LPGs), and the overall reduction of procurement prices by at least 2 times for lowest priced generics (LPGs) and 6 times for OBs. These governmental interventions resulted in a more effective medicine procurement in 2015 compared to 2010.

## 03 Post marketing Surveillance and costs of leading brands Of ciprofloxacin tablets marketed in urban Areas Of Mongolia

### Azjargal Ganbat,^1, 2^ Tsetsegmaa Sanjjav ^1,3^ Bruce Sunderland,^4^ Gantuya Dorj^5^ Gereltuya Dorj^1^

#### ^1^School of Pharmacy, Mongolian National University of Medical Sciences, S. Zorig street, 14210 Ulaanbaatar, Mongolia; ^2^Mongolian University of Pharmaceutical Sciences, Sonsgolon Road-5, Songinokhairkhan district, Ulaanbaatar 18130, Mongolia; ^3^Drug Research Institute, Monos group, Sonsgolon Road-5, Songinokhairkhan district, Ulaanbaatar 18130, Mongolia; ^4^Faculty of Health Sciences, Curtin University, Kent Street, Bentley, Perth, Western Australia 6102; ^5^School of Public Health, Mongolian National University of Medical Sciences, S. Zorig street, 14210 Ulaanbaatar, Mongolia

##### **Correspondence:** Gereltuya Dorj (gereltuya.dorj@mnums.edu.mn)

Postmarketing surveillances are crucial to obtain data of a product after it had been granted marketing authorization. The information is useful for product improvement, development of standards and regulations. The study aimed to examine *in vitro* quality control tests and estimate the cost of five brands of ciprofloxacin hydrochloride 500 mg tablets available in Mongolia. The quality control parameters of five different brands of ciprofloxacin hydrochloride 500 mg tablets were tested and evaluated against British Pharmacopoeia (BP) and Mongolian National Pharmacopoeia (MNP). The cost of five leading brands of ciprofloxacin hydrochloride 500 mg tablet was obtained from the selected retailers and wholesalers by using a WHO standardized methodology. A Median Price Ratio (MPR) was calculated in order to compare the wholesaling and retail costs of ciprofloxacin tablets with their counterparts reported by the Management Sciences for Health (MSH). All brands were in compliance in terms of weight variation (0.8%-3.4%) and friability (0.6-1.5). Assay content indicated that all samples were within approved limits [Brand C -98.4%, Brand B -100.3%, Brand D -99.1%, Brand A-101.5%, Brand C-102.8%]. However, disintegration and dissolution tests were non-compliant for Brand B and C. The mean cost of ciprofloxacin tablets was 0.12 US$ (0.05-0.33US$) at wholesaler and 0.13 US$ (0.07-0.24US$) at the retail level. When compared with international price, an MPR of 1.4 for wholesale and MPR of 2.6 for retail level was obtained. The costs of Brand B and C were twice at the wholesale level and up to 5 times higher than in other countries. Only 60% of sampled products met the BP and MNP requirements. More significantly, low quality ciprofloxacin tablets were more expensive than those at the international market. Regular post marketing studies would assure the quality and safety of ciprofloxacin, as well control the cost of ciprofloxacin in Mongolia.

## 04 Analysis of Drug Availability and Inventory Control in Health Centers,Province Yogyakarta, Indonesia

### Satibi Satibi, M. Rifqi Rokhman, Hardika Aditama, Ika Kartini., Rini Ambarsari, Fajar Pramesti

#### Departemen Farmasetika, Faculty of Pharmacy Universitas Gadjah Mada, Yogyakarta

##### **Correspondence:** Satibi Satibi (satibi@ugm.ac.id)

Drug availability is very important and closely related to service quality. The availability of medicine is the main pillar in creating patient satisfaction, doctors and pharmacy staff, so patients will get drugs according to medical needs. The availability of safe drugs makes the budget more efficient and effective. The purpose of this study was to study drug research and control conducted in health centers, DI Yogyakarta Province. This research is a descriptive non-experimental study. The study was conducted in 12 health centers in 3 districts and cities, namely Sleman, Bantul and Yogyakarta. Data was taken retrospectively by tracking drug management documents as secondary data, also carried out observations and interviews for pharmacists. The indicators taken are drug availability, inventory turnover ratio, empty stock, excess stock, safe stock, lack of stock, dead stock, expired drug value, damaged drug value and time of drug emptiness. This study received ethical permission from the Faculty of Dentistry, Universitas Gadjah Mada, No: 001426 / KKEP / FKG-UGM / EC / 2018. Data analysis was carried out descriptively by calculating indicator values ​​by calculating and comparing the standards for each health centers. Availability of medicines in health centers in DI Yogyakarta province varied in value, Sleman District Public Health Center was 18.36 months, in Bantul was 25.82 months and Yogyakarta City was 69.58 months. In controlling the drug in DIY, there is an excessive stock for each drug with a value of 22.59%, 23.99% and 33.72%. Factors that contributed towards excessive stock because the drugs that experience dead stocks are quite high. Expired drug values ​​varied between 1.14%, 2.23% and 4.84% of total inventory. Drug emptiness still occurs even though it is small (0.48%), with the length of time the drug emptiness ranges from 1.42, 5.76 and 15.76%. however there is still a lack of drug stocks of 0.36%, 0.45% and 10.48%.The availability of drugs in the DI Yogyakarta provincial health center is excessive with the drug control system not yet implemented effectively and efficiently.

## 05 Do Community Pharmacists in Malaysia Provide Pharmaceutical Care?

### Peng Yeow Loh^1^, Siew Siang Chua^1^, Mahmathi Karuppannan^2^

#### ^1^ School of Pharmacy, Taylor's University, Subang, Selangor, Malaysia; ^2^ Faculty of Pharmacy, Universiti Technologi MARA, Malaysia

##### **Correspondence:** Peng Yeow Loh (lohpengyeow@gmail.com)

Since the introduction of pharmaceutical care philosophy internationally by Hepler and Strand in 1990, community pharmacists worldwide, including in Malaysia, have been realigning their roles from being product-focus to patient-orientated, to achieve definite outcomes that could improve a patient's quality of life. The main aim of this research was to assess the practice of pharmaceutical care by community pharmacists in Malaysia. The study results would aid healthcare stakeholders in the planning of the healthcare system in Malaysia. A cross-sectional observational study was conducted where data was collected from community pharmacists throughout Malaysia using an online questionnaire. The questionnaire was developed based on the five principles of practice of pharmaceutical care as defined by the American Pharmacists Association (APhA). Of the 420 respondents, 53.3% claimed that they have been providing pharmaceutical care services to their patients with chronic diseases. Based on the five practice principle of pharmaceutical care, 98 respondents (23.3%) had patient’s data collection, 78 (18.6%) conducted medical information evaluation, 39 (9.3%) were involved in formulating drug therapy plan, 19 (4.5%) in implementing drug therapy plan, and 77 (18.3%) in monitoring and modifying the plan. A majority of the respondents felt that government policy was a barrier to the implementation of pharmaceutical care services in community pharmacy settings. These included a lack of separation between prescribing and dispensing (84.0%), and government’s initiative for patients to obtain their prescribed medicines from community pharmacies (83.8%). The present study shows that the provision of the pharmaceutical care services by community pharmacists in Malaysia is minimal. Therefore, there is an urgent need for government healthcare policies to act as a catalyst to optimise the utilisation of healthcare providers in the country to achieve a better national healthcare transformation.

## 06 Views of Community Pharmacists on Barriers to Sustain the Weight Management Services from Community Pharmacies: A Qualitative Study Malaysia

### Rohit Kumar Verma^1,4^, Thomas Paraidathathu^2^, Nur Akmar Taha^3^, Wei Wen Chong^4^

#### ^1^Department of Pharmacy Practice, School of Pharmacy, International Medical University 57000 Kuala Lumpur, Malaysia; ^2^Faculty of Health and Medical Sciences Taylor’s University, 47500 Subang Jaya, Malaysia; ^3^Faculty of Pharmacy, Cyberjaya University College of Medical Sciences, 63000, Cyberjaya, Selangor, Malaysia; ^4^ Faculty of Pharmacy, Universiti Kebangsaan Malaysia, 50300, Kuala Lumpur, Malaysia

##### **Correspondence:** Rohit Kumar Verma (rohitkumar_verma@imu.edu.my)

Community pharmacists (CP) are delivering an increase number of extended services and are potential well placed to contribute to obesity management strategies. No study to date have investigated the views of CP in Malaysia about their barriers in weight management.

Qualitative, face-to-face, structured interview were undertaken with 24 community pharmacists in Klang valley, Malaysia. The main barriers described included: (i) initiation of weight topic discussion with overweight/obese subjects; (ii) a lack of private consultation room; (iii) a lack of remuneration and time (iv) a lack of qualified staff and lack of training; (v) and a lack of public awareness. Almost all participants revealed difficulty to introduce the potentially sensitive issue of weight with their clients for fear of causing offence. They would hence normally wait for their clients to take the initiative, or bring out the topic in the context of other health conditions, especially diabetes, hypertension, or osteoarthritis. Therefore, a few participants identified a lack of consultation room to discuss the topic privately as a barrier to involve in weight management service. One of the barriers perceived by participants was a lack of financial reimbursement. A lack of time also posed a barrier to the administration of weight management services among some participants. This was especially true for community pharmacies that were always crowded with customers or community pharmacies that were operated with only one or two pharmacists. A lack of qualified staff in the pharmacy was also cited. Qualitative study findings showed that the community pharmacists have good understanding on the obesity management and they were positive towards providing the Weight Management Service (WMS). However, community pharmacists emphasised on monitoring, follow-up and referral to other healthcare professional for effective management of obesity issues in Malaysia.

## 07 Evaluation of Home Medication Review by Community Pharmacists (HMR-CP) for Patients with Type 2 Diabetes Mellitus (T2DM)

### M Rozaini Rosli ^1^Chin Fen Neoh^1^, Mahmathi Karuppannan^1^, W Nazariah W Hassan^2^, Mahani Mahmud^2^, Afifah Rahimi^2^, David Bin-Chia Wu^3,4^.

#### ^1^Faculty of Pharmacy, Level 11, FF1 Building, UiTM Puncak Alam Campus, Selangor, Malaysia ^2^Bandar Pasir Mas Health Clinic, Jalan Hospital, Pasir Mas, Kelantan, Malaysia; ^3^School of Pharmacy, Monash University Malaysia, Bandar Sunway, Selangor, Malaysia; ^4^Asian Centre for Evidence Synthesis in Population, Implementation and Clinical Outcomes, Health and Well-Being Cluster, Global Asia in the 21st Century Platform, Monash University Malaysia, Bandar Sunway, Selangor, Malaysia;

##### **Correspondence:** M Rozaini Rosli (zaini_0601@yahoo.com)

Diabetes is one of the chronic diseases that needs patient’s commitment and understanding of disease management. This trial aimed to evaluate the effectiveness of home medication review by community pharmacists (HMR-CP) programme in optimising diabetes care. A randomised controlled trial was conducted in Bandar Pasir Mas Health Clinic, Kelantan, Malaysia from March to December 2018. A total of 166 patients with T2DM were randomly assigned to either control or intervention groups. Seven community pharmacists were trained to perform HMR and they visited patients’ house every three months (i.e. baseline, 3-month and 6-month). Clinical outcomes, anthropometric data and humanistic outcomes were determined using intention-to-treat population. For the intervention group, drug-related problems (DRP) were classified according to the Pharmaceutical Care Network Europe Foundation (PCNE). Medication adherence was determined based on pill counting adherence ratio (PCAR) and the total cost of drug wastage was then calculated accordingly. Incidence of hospitalisation was documented as well. All data collected were then analysed using SPSS. No significant difference was seen in demographic and anthropometric data at baseline between the two groups except for fasting blood glucose (FBG). Significant reduction in glycated haemoglobin (HbA1c) (p < 0.001) and FBG (p = 0.002) within the intervention group across the three time-points were reported. After adjusting for baseline body mass index (BMI), FBG and demographic variables, the reduction in HbA1c (β = -0.386, 95%CI: -0.647 to -0.126, p = 0.004) and FBG (β = -0.038, 95%CI: -0.069 to -0.008, p = 0.015) remained significant over time according to the intervention assignment (i.e. in the HMR-CP group) at 6-month follow-up. A similar observation was noted in diastolic blood pressure (DBP) and total cholesterol (TC) but not in systolic blood pressure (SBP), high-density lipoprotein (HDL) and anthropometric parameters. Both utility value and Michigan Diabetes Knowledge Test (MDKT) scores increased significantly over time according to the intervention assignment. The most common type of DRP was “effect of drug treatment not optimal” (n = 67, 98.5%). Within the intervention group, significant changes in PCAR (p<0.001), the cost of medication wastage (p = 0.014) and the number of DRP (p<0.001) were noted. HMR-CP had significantly improved the T2DM patient’s glycaemic control, quality of life, medication adherence and knowledge while reducing the number of DRP and the cost of the medication wastage. However, the impact of HMR-CP on certain clinical parameters, and anthropometric parameters, remain inconclusive and this merits for further investigation.

## 08 Provision of Professional Pharmacy Services amongst Community Pharmacists towards Cardiovascular Diseases Secondary Prevention

### Ren Ee Teh, Adliah Mhd Ali (adliah@ukm.edu.my)

#### Faculty of Pharmacy, Universiti Kebangsaan Malaysia, Kuala Lumpur, 50300 Malaysia

This study aims to identify the scope of provision for Professional Pharmacy Service (PPS) amongst community pharmacists in supporting cardiovascular diseases (CVD) secondary prevention in Kuala Lumpur and to identify pharmacy and/or pharmacist’s characteristics which predict PPS provision and CVD secondary prevention. This is a cross-sectional self-administered survey conducted among the community pharmacists in Wilayah Persekutuan Kuala Lumpur from April to June 2019. A total of 132 community pharmacists participated in this study. Respondents were mostly female (66.7%) with the mean age of the respondents 33.06 ± 7.3 years old. Only eight respondents held academic qualification higher than bachelor degree in Pharmacy. Approximately 84.9% of the participants have working experience between 1-5 years (56.1%) to 6-10 years (28.8%) respectively in a retail pharmacy setting. Approximately three quarters of community pharmacists (72.7%) engaged in continuous professional development (CPD) activities within the past six months. All respondents reported to offer blood pressure and blood glucose testing services to clients. Majority of the respondents 81.8% offered blood cholesterol testing service, followed by 69.7% of respondents providing smoking cessation service. Weight management service was the least likely to be offered by community pharmacists amongst the five professional pharmacy services (PPS), with only 65.2% of the respondents provided such service. The majority of respondents reported on providing counseling on medicines and monitoring of medicine related side effect (90.9% and 86.4%, respectively) for clients with established CVD. Other monitoring activity was less likely to be provided such as monitoring for changes in lifestyle (83.3%). Almost all respondents were reportedly actively providing information regarding CVD condition(s) and advice on lifestyle modification (89.4% and 92.4%, respectively). However, it appeared that most respondents provided at least four types of activities (86.4%). Only a small fraction of respondents (24.2%) has worked with other healthcare professionals with regards to client CVD management. Factors associated with CVD provision included the provision of PPS, frequency of working with other healthcare professionals, number of pharmacist(s) or supporting staff(s) on duty, characteristics of pharmacists, pharmacy infrastructure and pharmacist resource adequacy. However, only CPD activities, documentation system in place and working with other healthcare professionals were found to influence CVD support. Most of the pharmacists were involved in the provision of CVD secondary prevention, however, a more structured intervention needs to be developed in expanding the role of the community pharmacist in CVD secondary prevention.

## 09 Impact of Guidelines Non-Adherence on Mortality among Patients Managed for Acute Ischemic Stroke

### Mohammed Mustapha^1,2^, Hadzliana Zainal^1^, Balamurugan Tangiisuran^1,3^, Sabariah Noor Harun^1^, Irene Looi^4^, Norsima Nazifah Sidek^5^, Khairul Azmi Ibrahim^5^, Loo Keat Wei^6^, Lee Keng Yee^7^, Zariah Abdul Aziz^8^

#### ^1^ School of Pharmaceutical Sciences, Universiti Sains Malaysia, 11800 USM, Pulau Pinang, Malaysia; ^2^ Department of Clinical Pharmacy and Pharmacy Practice, Ahmadu Bello University Zaria, Nigeria; ^3^ National Poison Centre, Universiti Sains Malaysia, 11800 USM, Pulau Pinang, Malaysia; ^4^ Clinical Research Centre, Hospital Seberang Jaya, Pulau Pinang, Malaysia; ^5^ Clinical Research Centre, Hospital Sultanah Nur Zahirah, Terengganu, Malaysia; ^6^ Department of Biological Science, Faculty of Science, Universiti Tunku Abdul Rahman, Kampar Campus, Perak, Malaysia; ^7^ National Clinical Research Centre, Kuala Lumpur, Malaysia; ^8^ Clinical Research Centre, Hospital Sultanah Nur Zahirah, Terengganu, Malaysia

##### **Correspondence:** Mohammed Mustapha (macreener88@gmail.com)

Stroke is a leading cause of death worldwide. The cases of acute ischemic stroke are on the increase in the Asia Pacific particularly in Malaysia. Guidelines for managing stroke have been recommended by various health organizations, but adherence to such guidelines and impact on patient outcomes including mortality are rarely explored. The aim of this study is to evaluate the impact of guideline non-adherence on mortality among patients managed for acute ischemic stroke. All stroke patients diagnosed with first-ever acute ischemic stroke (AIS) enrolled in the multiethnic National Neurology Registry (NNEUR) and followed up for 6 months were included in this study. Patients’ baseline clinical characteristics, risk factors, neurological findings, treatments, complications and outcome (survival status) data were reviewed. Nonadherence rate (NAR) was calculated based on the key performance indicators (KPI) of the NNEUR. Data were analyzed using SPSS version 20.0. *p* ≤ 0.05 was considered statistically significant. A total of 579 first ever AIS patients were included in the final analysis. The overall mortality was recoded as 23 (4.0%) with a median (interquartile) age of 65 (20) years. Majority of the patients that died were PACI (43.5%) and TACI (26.1%) stroke types. NAR was more common with DVT prophylaxis (82.8%), and anticoagulant for AF (49.8%). The Cox model showed mortality and OCSP groups directly correlated with NAR (*p*<0.05). The median NAR was 2 (range, 0 - 5). The risk of death was found to be more likely among patients with NAR > 2 (*p*= 0.05), POCI and LACI clinical groups (*p*=0.023). Oxfordshire Community Stroke Project (OSCP); Nonadherence rate (NAR); Total anterior circulation infarct (TACI); Partial anterior circulation infarct (PACI); Lacunar infarct (LACI); Posterior circulation infarct (POCI); * significant at p ≤ 0.05 using Chi-square. Guideline adherence in acute ischemic stroke management was sub-optimal. The nonadherence as well as patients’ risk factors were associated with mortality. These findings suggest continuous quality assessment and implementation of guidelines-based practice in stroke management

## 10 Association of Clinical Characteristics and Management of Critically Ill Sepsis Patients on Mortality Risk Factors and Length of Stay: (Predictors And Outcomes)

### Khalid A. Al-Sunaidar^1^, Noorizan Abd Aziz^2^, Yahaya Hassan^2^

#### ^1^Department of Pharmacy Practice, Faculty of Pharmacy, Universiti Teknologi MARA (UiTM), Puncak Alam,Selangor, Malaysia; ^2^Department of Pharmacy Practice, School of Pharmacy, Management and Science University, Shah Alam,Selangor, Malaysia

##### **Correspondence:** Khalid A. Al-Sunaidar (ksunaidar@gmail.com)

Sepsis is a leading cause of death in intensive care units (ICUs). It is also associated with long term morbidity and healthcare cost. The appropriate management is of greatest importance. Factors related to mortality include time to start antibiotics, infection control, and fluid infusion, in addition to factors intrinsic to patients, such as age and comorbidities. The aim of this study was to determine the association of characteristics and management of sepsis ICU adults’ patients on the predictors of 28 mortality and ICU length of stay (ICU-LOS) with outcomes. A retrospective cohort study conducted in tertiary ICU hospital in Selangor. 228 adult patients’ files with sepsis met the inclusion study criteria were reviewed. Univariate and multivariate (MVA) logistic & cox regression modelling were performed to detect relationship between patient characteristics and management (CM) with ICU-mortality. Also, univariate and (MVA) linear regression were used for determining association between (CM) and (ICU-LOS). Out of 228 ICU adult's patients 193(84.6%) were died with 119 (52.2%) male. The mean ICU-LOS was 9.86 days. The male gender [HR 1.38 (95% CI 1.032-1.860, *P*= 0.03)], surgery in ICU [HR 1.53 (95% CI 1.131-2.078, *P*= .006)], community acquired infection [HR 1.39 (95% CI 1.041-1.854, *P*=0.026)], mechanical ventilation duration 1-3 days [HR 40.52 (95% CI 5.383- 305.076, *P*<0.01)], 4 organs dysfunction [HR 1.820(95% CI 1.067-3.106, *P*=0.028)], *Acinetobacter baumannii* (AC) MRO infection [HR 1.802(95% CI 1.200- 2.706, *P*=0.005)], creatinine clearance on day-3 [HR 1.04(95% CI 1.011- 1.071, *P*= 0.007)] and DVT disease [HR 1.676 (95% CI 1.225- 2.294, *P*=0.001)] all were mortality risk factors. While in MVA cox regression only the 4 organs dysfunction [HR 0.128 (95% CI 025 -.654, *P*= 0.014)] and AC MRO infection [HR 0.102(95% CI 013 -.780, *P*= 0.028)] were protective risk factors. On the other hand, in MVA linear regression five variables were more likely to be associated as predictors for the increment of ICU-LOS (R^2^=0.478); Intermittent Dialysis (95% CI 1.776-6.464, *P*= 0.001), *Enterococcus faecalis* infection (95% CI 0.412- 7.713, *P*=0.029), AC.MRO infection (95% CI 0.064- 5.044, *P*= 0.044), DVT disease (95% CI 0.572- 4.247, *P*= 0.010) and surgery as source of infection (95% CI 0.408- 4.045, *P*= 0.017). The risk of mortality was high among this cohort sepsis ICU patients. The hospital initiative to reduce the emergence of multiple resistant microorganisms and antibiotics stewardship programs would improve the clinical outcomes and lessen ICU-LOS.

## 11 Economic Evaluation of Echinocandins for the Treatment of Candidaemia and Invasive Candidiasis: A Systematic Review

### Mohamed Abdul Hameed^1^, Nur Fatihah Binti Shaari^1^, Mahmathi Karuppannan^1^, Hasnah Ismail^1^, Yuet Yen Wong^2^, Chin Fen Neoh^1^

#### ^1^ Faculty of Pharmacy, Universiti Teknologi MARA (UiTM), Selangor Branch, Puncak Alam Campus, 42300 Puncak Alam, Selangor, Malaysia; ^2^ Faculty of Pharmacy, Universiti Teknologi MARA, Penang Branch, Bertam Campus, 12300 Kepala Batas, Penang, Malaysia

##### **Correspondence:** Mohamed Abdul Hameed (pharmahameed@gmail.com)

Candidaemia and invasive candidiasis (IC) are increasingly common among the critically ill and immunocompromised patients. Whilst echinocandins have been recommended as the first-line treatment and are associated with favourable safety profiles, however, they are expensive. Many economic evaluations published in this area; however no systematic review with methodological appraisal has been performed. The aim of this study was to identify and critically appraise the full economic evaluation of echinocandins for the treatment of candidaemia and invasive candidiasis. A detailed search of the literature was conducted for peer reviewed studies in multiple databases (i.e. PubMed, SCOPUS®, Cochrane, Web of Science, EconLit, Heoro.com and NHS EED). The characteristics of each included studies (i.e. country, study year, year of costing, type of currency, type of economic evaluation, objective, study perspective, time horizon, comparators, cost components, outcome measure, sensitivity analysis, and economic findings) were summarised. The reporting quality of the included studies were appraised using the 24-item Consolidated Health Economic Evaluation Reporting Standards statement (CHEERS checklist) and the methodological quality assessment was conducted using 10-item Drummond checklist. A total of 1194 articles were identified and 17 were included in the final review. The studies were either cost-minimisation analysis (n=9, 53%) or cost-effectiveness analysis (CEA) (n=8, 47%). Most of the reported studies employed decision analytical models. All studies reported cost per patient while several CEA also included the incremental cost per life year (LY) gained/saved or incremental cost per treatment success. The incremental cost-effectiveness ratio (ICER) per treatment success ranged from US$41,020 to US$147,207 and the ICER per LY gained ranged from US$244 to US$16,322. In general, all economic studies had a good reporting and methodological quality. Echinocandins were found to be cost-effective intervention in treating infections due to both *albicans* and non-*albicans Candida* species as compared to their alternatives.

## 12 Self-Management Knowledge And Its Contributing Factors: A Cross-Sectional Survey Among Asthmatic Patients In Hospital Sultanah Bahiyah, Alor Setar

### Rifqa Danisha Nadhrah Ramlan^1^, Wan Asma Najiha A Raman^1^, Syazani Salman Radzaini^1^, Norazila Abdul Ghani^2^, Qi Ying Lean^1,3^, Chin Fen Neoh^4,5^, Yuet Yen Wong^1^

#### ^1^Faculty of Pharmacy, Universiti Teknologi MARA, Cawangan Pulau Pinang, Kampus Bertam, Penang, Malaysia; ^2^Pharmacy Department, Hospital Sultanah Bahiyah, Alor Setar, Malaysia; ^3^Vector-Borne Diseases Research Group (VERDI), Pharmaceutical and Life Sciences CoRe, Universiti Teknologi MARA, Shah Alam, Selangor, Malaysia; ^4^Faculty of Pharmacy, Universiti Teknologi MARA, Cawangan Selangor, Kampus Puncak Alam, Selangor, Malaysia; ^5^Collaborative Drug Discovery Research (CDDR) Group, Pharmaceutical and Life Sciences Community of Research, Universiti Teknologi MARA, Shah Alam, Selangor, Malaysia

##### **Correspondence:** Yuet Yen Wong (yuetyen@yahoo.com)

Asthma affects two-million people in Malaysia. in depth exploration given that it plays integral role in achieving good asthmatic control. This study therefore aimed to: (1) assess self-management knowledge and asthma control among asthmatic patients attending Hospital Sultanah Bahiyah (HSB), Alor Setar; (2) explore factors affecting asthma self-management knowledge; (3) determine the relationship between self-management knowledge and level of asthmatic control. A cross-sectional survey, using pre-validated questionnaire, was conducted over 4 months (November 2018 - February 2019) at outpatient pharmacy department (OPD) in HSB. Convenience sampling method was used. Patients who fulfilled study inclusion and exclusion criteria were consented to the study. The self-management knowledge score was calculated based on correct responses provided. The score was converted into percentage with more than 75% deemed as good knowledge. All data were analysed using IBM statistical package for social sciences (SPSS) version 20 with a priori p < 0.05 considered statistically significant. A total of 274 questionnaires were completed and returned. The median asthma self-management knowledge score was 6 [Interquartile range (IQR) 2]. Only 17.9% of the respondents had good knowledge, 60.9% had adequate knowledge, and 21.2% had poor knowledge. Of all participants, 75.2% stated erroneously that asthma can be cured, 46.7% were confused about control and rescue anti-asthmatic medications and only 51.1% could answer correctly that exercise improves lung capacity. Our findings suggested that respondents with a high education level (p = 0.01) and had previously attended counselling (p = 0.005) tend to have higher asthma self-management knowledge scores. In addition, respondents with longer duration of asthma diagnosis were found to have better self-management knowledge scores (r = 0.136, p = 0.024). No significant differences in self-management knowledge scores were observed in other sociodemographic factors (i.e. age, gender, occupation and smoking status). We observed a significant positive correlation between asthma self-management knowledge scores and asthma control (r = 0.143, p = 0.018). The level of asthma self-management knowledge among adults attending HSB was adequate with several knowledge gaps remained. Healthcare professionals need to address such shortcoming given the findings from this study suggested that patients with greater self-management knowledge are more likely to achieve better asthma control.

## 13 A Comparison of the Incidence and Hazard Ratio of Diabetes Mellitus among Statin User Groups in a Malaysian Healthcare Setting

### Md Shariff A^1^, Karuppannan M^2^, Gnanasan S^2^ and A. Aziz N^3^

#### ^1^Department of Pharmacy, Hospital Tengku Ampuan Rahimah, Klang, Selangor, Malaysia; ^2^Department of Pharmacy Practice, Universiti Teknologi MARA, Puncak Alam Campus, Shah Alam, Selangor, Malaysia; ^3^Department of Pharmacy Practice, Malaysian Science University (MSU), Shah Alam Campus, Selangor, Malaysia

As certain conventional medications are becoming increasingly common among the current generation, their previously unexplored side effects and long-term effects have started to become a subject of scrutiny, and the link between these medications and long-term effects such as the development of diabetes, is starting to be explored. There has recently been a massive discovery in terms of the possible contribution of statins in the development of diabetes mellitus. This discovery has led to many subsequent debates and rumours or uncertainties over the safety and necessity of statins. As the discovery of the relationship between statins and Diabetes is still relatively new, there has yet to be extensive research exploring on the more specific details of the association. The individual risk of statin types have been explored but not extensively, specifically in Malaysia. To investigate the incidence of new-onset diabetes mellitus and to compare the risk of development of new-onset diabetes mellitus among different statin user groups or cohorts in a Malaysian healthcare setting. This was a retrospective cohort study. 507 patients without diabetes mellitus who were given statin treatment in Hospital Sungai Buloh were retrospectively enrolled and were subsequently divided into three cohorts according to the types of statin given. The mean duration of this study was 32.5 months (±18.1). The minimum duration of study was 2.9 months, while the maximum duration was 87.9 months. The incidence of DM was found to be highest in the simvastatin user group (20 of 114: 17.5%), followed by the mixed statin user group (35 of 213: 16.4%), and lovastatin (28 of 172: 16.3%), p=0.956. As the p value was >0.05, it was found that there was no significant difference in the incidence of DM among the three statin user cohorts. The risk of DM was found to be the highest in the Lovastatin user group compared to the mixed statin user group (Hazard ratio=1.88, p=0.017) followed by the Simvastatin user group (Hazard ratio=1.87, p=0.029). The risk of new-onset DM from lovastatin alone or simvastatin alone was significantly greater than those of mixed statin users, in which the statin type was switched at least once throughout the study, while the risk of new-onset DM from lovastatin and simvastatin were almost the same. There was no significant difference in the incidence of DM among the three statin user cohorts.

## 14 The Use of Herbal and Dietary Supplements among Elderly in Puncak Alam, Selangor, Malaysia

### Muhammad Helmi Zaini, Mohd Shahezwan Abd Wahab

#### Department of Pharmacy Practice, Faculty of Pharmacy, Universiti Teknologi MARA (UiTM), 42300 Bandar Puncak Alam, Selangor, Malaysia.

##### **Correspondence:** Mohd Shahezwan Abd Wahab (mohdsh2790@puncakalam.uitm.edu.my)

The use of herbal and dietary supplement (HDS) has been shown to be prevalent in many countries. Despite being normally perceived as safe, HDS may produce adverse effects, and can potentially interact with conventional medicines. There is a concern about HDS use in the elderly population due age-related physiological changes in their body. The pattern of use of HDS among elderly population in Malaysia is limitedly known. This study aims to investigate the prevalence of and pattern of HDS use among a sample of elderly in Puncak Alam, Selangor. A cross-sectional survey was conducted between March and May 2019 among elderly individuals aged ≥ 60 in Puncak Alam, Selangor. Elderly individuals with cognitive impairment or those who do not understand Malay or English languages were excluded. Data was collected using a pre-validated questionnaire. Overall, 336 elderly responded to the survey, meeting the minimum recommended sample size of the study. Overall, 45.8% (154/336) of respondents were using at least one type of HDS at the point of survey. The prevalence of use of at least one type of dietary supplement and herbal medicine was 42.6% (143/336) and 11.3% (38/336), respectively. HDS non-users mostly “prefer to use modern medicines” (62.6%, 114/182), and are “not interested in using HDS” (59.3%, 108/182). Almost half (49.4%, 76/154) of HDS users have been diagnosed with a medical condition. About 38% (59/154) of them were also using at least one prescription drug. Multivariate analysis showed that having good – excellent perceived health (adjusted OR = 2.666, 95% CI = 1.592 – 4.464), a history of sickness in the past one month (adjusted OR = 2.500, 95% CI = 1.426 – 4.383), having lower body mass index (adjusted OR = 0.937, 95% CI = 0.887 – 0.990) were predictors of HDS use. Only a small percentage of them (25/154, 16.2%) informed their HDS use to healthcare providers. Since many elderly individuals used HDS, healthcare providers should be more vigilant about this issue. There is also need for on-going campaigns to educate the public about HDS’ benefits and risks

## 15 ABC-VEN Analysis on Cardiovascular Medicines Used at The Tertiary Hospital in Mongolia

### Dulmaa Lkhagvasuren^1^, Enkhjargal Dorjbal^2^

#### ^1^ School of Pharmacy, Mongolian National University of Medical Sciences; ^2^ School of Pharmacy, Mongolian National University of Medical Sciences

##### **Correspondence:** Enkhjargal Dorjbal (enkhjargal.d@mnums.edu.mn)

As cardiovascular diseases are the great public health problem globally, the management of cardiovascular medicines should not be out of attention. The ABC, VEN and ABC-VEN matrix analysis are carried out to identify the categories of cardiovascular medicines which are in the Essential Medicine List of Mongolia. Cross sectional design was used to carry out this study. The study was conducted at the Third State Central Hospital of Mongolia in FY 2018-2019. The annual cardiovascular medicines comsumption and expenditure were collected. ABC, VEN and ABC/VEN matrix analysis were used. Total 33 cardiovascular medicines which are in the Essential Medicine List of Mongolia were used during FY 2018-2019. In the ABC analysis 5 (15.2%), 7 (21.2%) and 21 (63.6%) drugs were classified as A, B and C category respectively. Whereas in the VEN analysis 23 (70%), 8 (24%) and 2 (6%) drugs were considered Vital, Essential and Non-essential items respectively. The ABC-VEN matrix analysis showed that category I, II and III contained 26 (78.8%), 6 (18.2%) and 1 (3%) drugs respectively. The results show that the medicines belonging to category I should be managed with great attention. The ABC, VEN and ABC-VEN matrix analysis should be carried out routinely in order to manage inventory effectively and properly in the hospitals.

## 16 Evaluation And Prevalence Of Pharmacovigilance Among Healthcare Professionals In Pakistan

### Qurratul-ain Leghari^1,2^, Muhammad Shahzad Aslam^3^, Neelum Malick^4^, Sadia Kashif^4^, Sharmeen Bawani^5^

#### ^1^ Department of Pharmaceutical Chemistry, Faculty of Pharmacy, Ziauddin University. Clifton Karachi, Pakistan; ^2^ Department of Pharmaceutical Chemistry, Faculty of Pharmacy, University of Karachi, Pakistan; ^3^ Department of Chemistry, Xiamen University Malaysia, Sepang, 43900, Malaysia; ^4^ Department of Pharmacology, Faculty of Pharmacy, Ziauddin University, Clifton Karachi, Pakistan; ^5^ Department of Pharmaceutics, Faculty of Pharmacy, University of Karachi, Pakistan

##### **Correspondence:** Muhammad Shahzad Aslam (aslam.shahzad@xmu.edu.my)

To increase the safe usage of medicines, it is important that these ADRs be reported. Many countries around the world have well established national Pharmacovigilance systems that depend upon reporting of ADRs by physicians and pharmacists. The investigation of the knowledge, attitudes, and perception of healthcare professionals towards the Pharmacovigilance framework and ADR reporting has already been explored in the developed nations yet there is an absence of studies in Pakistan. This study was carried out to gain insight into the knowledge, attitude, and perception of physicians, pharmacists, and nurses working in the private sector towards Pharmacovigilance and ADR reporting by conducting a questionnaire-based survey. Their knowledge of ADR reporting, the concept of Pharmacovigilance, the type of ADRs that are to be reported are discussed along with their attitude and perception as to how important ADR reporting is. The reasons and factors of under-reporting or not reporting ADRs are also evaluated. In order to achieve the research objectives, a total of 375 questionnaires were distributed out of which 125 (33.3%) for each category that was doctors, pharmacists and nurses respectively. The findings revealed that the majority of respondents (n = 205, 54.7 %) were not aware of the term and concept of Pharmacovigilance. Even though a majority of respondents (n=230, 61.3%) felt that ADR reporting is a professional obligation that needs to be fulfilled, only (n=155, 41.3%) healthcare professionals claimed to have been trained on how to actually report an ADR. This study and its findings established the absence of knowledge about Pharmacovigilance and ADR reporting among healthcare professionals.

## 17 Stakeholders of the Pharmaceutical Industry Perceptions on Medicine Price Transparency Practice in Malaysia Private Health Care Settings

### Nur Sufiza Bt Ahmad^1,2^, Ernieda Hatah^1^ & Mohd Makmor-Bakry^1^

#### ^1^ Discipline of pharmacy practice, Faculty of Pharmacy, Universiti Kebangsaan Malaysia, Jalan Raja Muda Abd Aziz, 50300, Kuala Lumpur; ^2^ Pharmaceutical Services Division, Ministry of Health Malaysia, 46350, Petaling Jaya, Selangor, Malaysia

##### **Correspondence:** Ernieda Hatah (ernieda@ukm.edu.my)

Price transparency in healthcare can be defined as readily available information on the price of healthcare services or medicines that, together with other information, helps define the value of those services or medicines.[1] While the policy makers are advised to achieve more transparent prices and contract structures for medicines to address disproportionate price differences within and between countries [2], pharmaceutical industries stakeholders’ view on medicine price transparency practice is still lacking. In Malaysia, the voluntary reporting of medicine prices have been started since 2011 [3], nevertheless only a few pharmaceutical companies were reported to declare their medicine price to the Pharmaceutical Service Department [4]. Since the effect of medicine price transparency depend critically on how prices are presented, it is important to gather the stakeholders of pharmaceutical industries’ insights on the potential benefits and threats of medicine price transparency practice to industries and market. Thus, this study aims to evaluate the stakeholders of the pharmaceutical industries’ perspective of medicine price transparency practice in the private healthcare system in Malaysia. This study was conducted as face-to-face, semi-structured interview. Respondents from four main stakeholders in the pharmaceutical industry which are the drug manufacturers or product holders, community pharmacists, general practitioners and private hospital pharmacists were recruited using purposive sampling. Using phenomenological study approach, interviews were conducted at respondents’ practices and were audio recorded with their consent. Data were transcribed verbatim and analysed using thematic analysis with Atlas.ti 8 software and categorised as strengths, weaknesses, opportunities and threats (SWOT). A total of 23 respondents were interviewed. Summary of the respondents’ background is presented in **Table 1**. SWOT analysis is summarized in **Table 2**. There were mixed perceptions on medicine price transparency practice in Malaysia. Although it was perceived to benefit the industries by standardizing the medicine pricing, reduce price variability, provide reference and control medicine price, there were concerned that the practice may affect stakeholders’ business and marketing strategy, reduce profit margin, increase general practitioner’s consultation fees and causing medicine price geographical discrepancies. The practice was viewed as an opportunity to disseminate the truth price information to consumer but it may give advantage to big pharmaceutical industries to cut competition with reduce price. Proper planning before implementing the compulsory price transparency practice is needed. Improper implementation may cause medicine price to increase or reduce business abilities of the pharmaceutical industries, hence affect public’s accessibility and affordability to medicine.

**Table 1 (abstract 17).** Summary of respondents’ demographics

No	Group	Participants	Gender	Years of Experience
1	*PI1*	Manufacturer/Product Holder	Male	30
2	*PI2*	Manufacturer/Product Holder	Male	30
3	*PI3*	Manufacturer/Product Holder	Male	20
4	*PI4*	Manufacturer/Product Holder	Female	28
5	*PI5*	Manufacturer/Product Holder	Male	30
6	*PI6*	Manufacturer/Product Holder	Male	6
7	*PI7*	Manufacturer/Product Holder	Male	23
8	*CP1*	Community Pharmacy	Female	22
9	*CP3*	Community Pharmacy	Female	10
10	*CP4*	Community Pharmacy	Female	19
11	*CP5*	Community Pharmacy	Female	18
12	*CP6*	Community Pharmacy	Female	16
13	*CP7*	Community Pharmacy	Male	6
14	*PH1*	Private Hospital	Female	19
15	*PH2*	Private Hospital	Female	28
16	*PH3*	Private Hospital	Female	12
17	*PH4*	Private Hospital	Female	17
18	*PH5*	Private Hospital	Female	18
19	*GP1*	Private Clinic	Female	10
20	*GP2*	Private Clinic	Male	28
21	*GP3*	Private Clinic	Female	15
22	*GP4*	Private Clinic	Male	16
23	*GP5*	Private Clinic	Male	22

**Table 2 (abstract 17).** SWOT analysis of respondents’ view of medicine price transparency practice

Internal Factor	Strengths	Weaknesses
	Standardize medicine price & reduce price variability	Reduce pharmaceutical industries’ profit margin, hence jeopardizing their survival
	Provide reference price to consumer and health care providers	Remove good price for certain facilities/ health care providers through abolishment of tier pricing and/or bonusing
	Increase consumer empowerment in value-based purchasing	Increase geographical pricing
External Factors	Opportunities	Threats
	Resolve consumer’s suspicion on industries for setting high mark-up for medicine price	Due to reduction in mark-up price, to ensure business survival, other related cost such as GP’s consultation fee may increase
	Prevent unreasonable increase in medicine price	Availability of reference price to other countries (External Reference Price, ERP) may reduce optimal monopoly price at home/country that needed it, for example, the least developed countries
	Strengthen the collaboration between pharmaceutical industries and the government	Reduce competition may cause market monopoly
	Promote healthy competition between the industries	Lack of regulation, increase the risk for price manipulation by the pharmaceutical industries
	Ease the process of itemized billing	Reduced profit margin, hence reduce investment in R&D, in turn, translate into fewer or less innovative new products
	Healthcare providers could focus more to service and treatment care	

## 18 Pharmaceuticals and public policy in the future of pharmacy: A review of the literature

### Raveena Amee Nagaria, Syed Shahzad Hasan, Zaheer Ud-Din Babar

#### Department of Pharmacy, University of Huddersfield, Queensgate, Huddersfield, HD1 3DH, United Kingdom

The pharmacy profession is changing and adapting its role within healthcare systems in response to changes in patient demographics worldwide. Funding priorities within the healthcare system are determined by evidence of improvement in clinical practice and cost-effectiveness of medication therapy and the delivery of services. To critically review literature relating to pharmaceuticals and public policy in the context of the future of pharmacy. Medline, Pubmed, Science Direct, Scopus and Springer Link electronic databases were searched for peer-reviewed literature using keywords such as “pharmacy practice” “future” “pharmaceuticals” “pharmaceutical policy” “healthcare” and “public policy”. Titles and abstracts were screened and data from eligible full-text articles dated from June 2009 to June 2019 was extracted. A total of 10 studies were included. Some studies highlighted the need for balancing expenditure between spending on pharmaceuticals and remuneration for pharmacy service delivery. This could be achieved by greater drug pricing transparency coupled with patient-centered, pharmacist-led services focused on medication therapy management. Other studies discussed the need to advocate and reconfigure the role of the pharmacist in policy documents, highlighting how pharmacists’ skills and expertise add value in patient-centred healthcare decision-making. Overall, the literature suggested the need for developing a model that could be used to drive changes in practice in the future. As pharmacy practice models are continuously evaluated, it is vital to explore how pharmaceuticals and public policy have an impact upon policy-making decisions within the healthcare system in the future.

## 19 Challenging Role of Pharmacists in Achieving Universal Health Coverage: A Qualitative Study in the Philippines

### Erwin Martinez Faller^1^, Jesa Madelo^2^, Edward Agravante Tolentino^3^

#### ^1^Research Management Center, Tagum Doctors College, Inc., Visayan Village, Philippines, 8100; ^2^Pharmacy Department, Tagum Doctors College, Inc., Visayan Village, Philippines, 8100; ^3^Supply Chain and Business Development, Regicon Healthcare Inc., Makati City, Philippines

##### **Correspondence:** Erwin Martinez Faller (erwinfaller1007@gmail.com)

Pharmacists are contributing in public health activities in low– and middle–income countries to facilitate strengthening of health equity in the country. The recent Universal Health Care (UHC) law or Republic Act No. 11223 calls for the participation of pharmacists in providing easier access to medicines. This study aims to identify the challenging role of pharmacists in achieving UHC in the Philippines. This study employed a qualitative phenomenological research design using face-to-face interviews conducted from May to July 2019. Purposive snowball sampling technique were used in 20 pharmacists from village pharmacies (*botika ng barangay*), public hospitals and regulatory agencies. The pharmacists narrated their experiences and barriers encountered in performing their role in attaining UHC through in-depth interviews, which were collected, transcribed and explored using the thematic analysis approach. The preliminary findings of the study revealed that pharmacists were involved in improving compliance to prescribed regimen, and accessibility to affordable, quality and cost-effective medications; monitoring patient’s treatment; and counseling patients and their caretakers on appropriate and rational drug therapy. Pharmacists also participated in public health activities such as prevention and control of non-communicable diseases through health education on promoting lifestyle changes and avoiding modifiable risk factors. Government hospitals initiated clinical pharmacy services to assign pharmacists in direct patient care. Barriers in UHC showed inadequacy of funding for medicine, facilities and services; underutilization of pharmacists; insufficient number of pharmacists employed in government and private institutions and competency in providing clinical pharmacy services. The recent passage of the UHC law has a major impact in the shift of the pharmacist’s role from a product-oriented practice to a patient-focused service. Thus, pharmacists are getting ready for their enhanced role in the health sector to attain health for all Filipinos.

## 20 Investigation of Adverse Drug Reactions Experienced by Smokers Attending Quit Smoking Clinics in Kuala Lumpur, Malaysia

### Zakiah Mohd Noordin^1^, Mahmathi Karuppannan^1^, Neoh Chin Fen^1^, Nor Haizan Ibrahim @ Ghazali^2^

#### ^1^Department of Pharmacy Practice, Faculty of Pharmacy, Universiti Teknologi MARA, Bandar Puncak, Alam, Selangor, Malaysia; ^2^Pharmacy Department, Klinik Kesihatan Kuala Lumpur, Jalan Temerloh, Taman Tasik Titiwangsa, 53200 Kuala Lumpuur

Utilisation of pharmacologic treatment in smoking cessation programme lead to highest quit rate. However, adverse drug reactions (ADR) associated with varenicline and nicotine replacement therapy may impair patient’s effort to quit. This research aims to investigate the ADRs experienced by smokers attending smoking cessation programme in Malaysia upon being prescribed with pharmacotherapy agents to quit smoking. This retrospective study was conducted in May 2019 at 4 quit smoking clinics (QSC) in Kuala Lumpur. A convenient sample of 285 smokers attended QSC between January 2016 to December 2018 was enrolled and categorized into 2 quit smoking status; successfully achieved 6-month abstinence and failed to achieve 6-month abstinence. Information on socio-demographics, smoking history, pharmacotherapy agent prescribed, and adverse drug reactions were collected from smoker’s medical records and analyzed by Pearson Chi-Square statistical test. The mean age of smokers is 48, with majority(37%) of the smokers age between 35 to 54 years old. 52% smokers are Malay, 33% are Chinese and 42% are Indians. 90% (256) of smokers are male. Of 285 smokers, 70 were prescribed nicotine gum and patch, 58 were prescribed varenicline alone, 55 were prescribed varenicline with nicotine gum, 51 were prescribed nicotine patch only, 27 were given nicotine gum only and 5 smokers were given combination of all three agents. ADR occurred in 139 out of 285 smokers (48.8%) and occurred mostly during the early stage of treatment; 2-4 weeks following initiation. Of 139 smokers experiencing ADR, 98 (70.5%) failed to quit while 41(29.5%) smokers successfully quit following recruitment. Highest ADR reported due to varenicline (42.9%), followed by nicotine patch (30%) and nicotine gum (27.1%). Throat irritation and nausea are the ADR reported highest for nicotine gum, irritation at site of infection for patient prescribed with nicotine patch and sleep problems in patients taking varenicline. There is no significant association between ADR experience and quit smoking status (p=0.461). The majority of adverse drug reactions reported were mild and patients often able to tolerate the ADRs following counselling from doctors or pharmacists. Thus, frequent monitoring of patient’s progress especially on ADR and consideration of additional or alternative treatments is important to ensure patients are able to quit smoking effectively.

## 21 Prevalence and Predictors of Herbal Medicines Use among Jordanian Adults

### El-Dahiyat F^1^, Rashrash M.^2^, Abuhamdah S.^2,3^, Abu Farha R^4^

#### ^1^College of Pharmacy, Al-Ain University of Science and Technology, Al Ain campus, United Arab of Emirates; ^2^College of Pharmacy, Al-Ain University of Science and Technology, Abu Dhabi campus, United Arab of Emirates; ^3^ Department of Biopharmaceutics and Clinical Pharmacy, Faculty of Pharmacy, The University of Jordan, Amman, Jordan; ^4^ Department of Clinical Pharmacy and Therapeutics, Faculty of Pharmacy, Applied Science Private University, Amman, Jordan

The aim of this study was to investigate the prevalence and determine factors, which predict the use of herbal medicine among Jordanian adults. A cross-sectional study was conducted on 378 older adults who were randomly selected from two different areas of Jordan. A questionnaire was used to gather the data. The data were described using the measures of descriptive statistics and analyzed via the Chi-square and multivariate logistic regression analysis. The majority of participants rated their health both excellent or very good (71.4%) and no significant association between the rated health and the usage of herbal medicines. About 80% of the study population did not report the presence of any chronic disease and no association between the presence of chronic illness and the use of herbal medicine. The most prevalent chronic disease among the study subjects was hypertension followed by diabetes (9.5%, 5.6% respectively) and there was a statistically significant association between type of chronic illness and the admitted use of herbal medicines. The herbal medicines were obtained mainly from Herbalist followed by the pharmacy (37.8, 23.0% respectively). Herbal medicine use was mainly recommended by family and friends in 39.7% followed by pharmacist and mass media (17.7%, 12.4% consecutively). Pharmacist and medical doctors were the most trusted persons in providing accurate information (24.6%, 23.3% correspondingly). The participants were disagree regarding the safety of herbal products and that herbal products are better than conventional medicines. The highest agreement was with the statement that says the herbals can maintain and promote health followed by their desire to know more about safety and efficacy of herbal medicines and the possibility of use herbals to treat illnesses (83.3%, 79.6%, and 77.8% respectively). There is a high use of herbal medicines in Jordan specially among hypertensive patients. Therefore, there is a need to amend the pharmaceutical policy to cover herbal medicines. There is also a need to establish effective health education programmes to discuss the benefits and risks of herbal medicine use with the aim of maximizing patient health outcomes.

## 22 Prevalence and Evaluation of Factors Associated with Depression Among Hemodialysis Patients

### Amjad Khan^1, 2, 3^, Amer Hayat Khan^1, 2^, Syed Azhar Syed Sulaiman^1^, Azreen Syazril Adnan^2^, and Saima Mushtaq^4^

#### ^1^Discipline of Clinical Pharmacy, School of Pharmaceutical Sciences, Universiti Sains Malaysia, Penang 11800, Malaysia; ^2^Chronic Kidney Disease Resource Centre, School of Medical Sciences, Health Campus, Universiti Sains Malaysia, Kubang Kerain 16150, Kelantan, Malaysia; ^3^Department of Pharmacy, Quaid-i-Azam University, Islamabad 45320, Pakistan; ^4^Health Care Biotechnology Department, Atta ur Rahman School of Applied Biosciences, National University of Science & Technology, Islamabad 44000, Pakistan

##### **Correspondence:** Amjad Khan (amjadkhan@qau.edu.pk)

Depression is one of the most common psychiatric disorders among hemodialysis (HD) patients but under-recognized. The present study was conducted with the aim to determine the prevalence and factors associated with depression among HD patients. Hospital Anxiety and Depression Scale (HADS) questionnaire was used to measure the depression level among 220 HD patients. This was a prospective follow-up study and the patients were asked to self-complete HADS questionnaire at three-time points: i) at baseline visit ii) after three months’ interval and iii) at six months’ interval (third follow up). In our study 157 (71.3%) patients suffered from depression at baseline, 169 (78.2%) on 2^nd^ evaluation and 181 (84.9%) on the final visit respectively. In multivariate logistic regression analysis, treatment given to patients at non-governmental organizations (NGO’s) running HD centers (OR= 0.347, p-value= 0.039) had statistically significant association with the prevalence of depression at final visit. Negative association of depression with dialysis therapy at NGOs running dialysis facilities is an indication of better depression management practices at these centers. This finding needs further investigation and therefore larger studies with longer follow-ups are warranted.

## 23 Self-Care Knowledge Among Type 2 Diabetes Mellitus Patients in Hospital Taiping, Malaysia

### Maisarah Mohamad Fadzil^1^, Muhammad Hadif Syahmi Mohd Akmal^1^, Yuet Yen Wong^1^, Chin Fen Neoh^2,3^, Qi Ying Lean^1,4^,

#### ^1^Faculty of Pharmacy, Universiti Teknologi MARA, Cawangan Pulau Pinang, Kampus Bertam, Penang, Malaysia; ^2^Faculty of Pharmacy, Universiti Teknologi MARA, Cawangan Selangor, Kampus Puncak Alam, Selangor, Malaysia; ^3^Collaborative Drug Discovery Research (CDDR) Group, Pharmaceutical and Life Sciences Community of Research, Universiti Teknologi MARA, Shah Alam, Selangor, Malaysia; ^4^Vector-Borne Diseases Research Group (VERDI), Pharmaceutical and Life Sciences CoRe, Universiti Teknologi MARA, Shah Alam, Selangor, Malaysia

##### **Correspondence:** Qi Ying Lean (leanqiying@yahoo.com)

Diabetes mellitus (DM) is a chronic metabolic disease associated with high morbidity and mortality. Type 2 DM (T2DM) is the most common type of DM, accounting for 90% of DM population. In Malaysia, DM has increased more than two-fold over the decades which needs serious actions to control the prevalence. Despite the availability of different hypoglycaemic agents, less than a quarter of patients in Malaysia achieved good glycaemic control. Even with the use of medications, patients self- management is essential as majority of diabetes care is practised by patients. Therefore, the main objective of our study was to determine the self-care knowledge among patients with T2DM in Hospital Taiping, Malaysia. A cross-sectional survey was conducted from December 2018 to February 2019. Adult patients with T2DM receiving treatment at Hospital Taiping were recruited through convenient sampling. A pre-validated, self-administered questionnaire was used to assess patient’s diabetes self-care knowledge. Total knowledge scores were calculated based on the correct responses provided. Chi-Square test was used to analyse the relationship of sociodemographic factors and the level of diabetes self-care knowledge. Data were analysed using SPSS with a p-value less than 0.05 was considered to be significant. A total of 148 adult patients diagnosed with T2DM participated in the survey. A total of 45.3% patients with T2DM in Hospital Taiping recorded a good knowledge score (i.e. score ≥ 70%) whereas more than half of patients (54.7%) had low diabetes self-care knowledge (< 70%). Most of the patients (98.6 %) knew that doctor make treatment planning by gathering data from the self-monitoring blood glucose and mostly (82.4%) thought that only the doctors should make plans on how a person with diabetes can achieve his/her health target goals. Education level (*p* = 0.041) was significantly associated with the level of self-care knowlege among patients. No significant difference in diabetes self-care knowledge scores observed in other sociodemographic factors [age (*p* = 0.542), gender (*p* = 0.275), ethicity (*p* = 0.179), and duration of diabetes (*p* = 0.068)]. Significant negative correlation was observed between self-care knowledge score and the fasting blood glucose (r = -0.264, *p* = 0.002). Several diabetes self-care knowledge gaps were identified from this study despite a proportion of respondents achieved high knowledge scores. The self-care knowledge among patients with T2DM is important to enable patients to practise self-care and to make good decisions in managing their diabetes.

## 24 Identifying The Predictors of The Effectiveness of Methadone Maintenance Therapy (Mmt) Among Opiate Dependent Patients Registered with Klinik Kesihatan Bayan Lepas and AADK Telok Bahang.

### Christina Malini^1^,Noor Azizah Binti Abdul Wahab^2^,Ahmad Fuad Shamsuddin^3^

#### ^1^ Hospital Seberang Jaya,Pulau Pinang; ^2&3^ School of Pharmaceutical Sciences,Royal College of Medicine,Perak.

##### **Correspondence:** Christina Malini (paarai03@yahoo.com)

Effectiveness of Methadone Maintenance Therapy (MMT) among opiate dependent individuals can be evaluated through changes observed in the quality of life (QOL) o these individuals after enrolling in the MMT programme. A study recently done in Klinik Kesihatan Bayan Lepas and AADK Telok Bahang proved that there is improvement in QOL of individuals after enrolling in MMT. Many factors affect the magnitude change in QOL. There is a big question mark where any predictors affect the effectiveness of MMT program in both centers. This study was undertaken to evaluate the predictors affecting the effectiveness of MMT programme and the QOL among opiate dependent individuals. In this study, 100 individuals from the Ministry of Health (MoH), Klinik Kesihatan Bayan Lepas (Bayan Lepas Health Clinic) and Agensi Anti-Dadah Kebangsaan (National Anti-Drug Agency) in Telok Bahang were involved. Percentage of daily attendance and drug urine results were collected during the study period. Subjects were predominantly of Malay ethnicity (82%) and aged between 51 to 60 years old (34%). The mean percentage of opiate relapse rate and compliance rate among MMT clients were 40.65% (±44.73) and 96.33% (SD±6.64) respectively. Pearson correlation was done to identify the relationship between compliance rate and opiate relapse rate and noted there was a significant negative relationship, r (100) = -2.3 and p =0.021. Age, education level and matrimonial status variables were tested through one- way ANOVA post hoc test. For social domain, secondary education was significant in the magnitude of score change in social domain, with mean difference of 14.75 and with a significance of 0.035 (p<0.05). Age and matrimonial status did not produce any significant magnitude of score outcome. Stepwise multiple linear regression analysis was performed and a significant regression equation was found (socioeconomic status) F(1)=5.402,p =0.022(p<0.05) with R^2^ of 0.044. Univariate analysis was done for physical and environmental domain, it was observed that the sex is associated with change in quality of life with the significance of 0.046, F=4.111 and 0.003, F=9.665 respectively. This study has identified the predictors that have highly contributed to improvement of QOL and strengthen the evidence of the effectiveness of MMT among MMT clients in Klinik Kesihatan Bayan Lepas and AADK Telok Bahang.

## 25 Knowledge, Attitudes and Perceptions towards Antimicrobial Use and Resistance Among Pharmacy Students in Universiti Teknologi MARA Malaysia

### Ahmad Fauzi Dali, Norfarahin Abd Patah, Noorizam Ibrahim

#### Department of Pharmacy Practice, Faculty of Pharmacy, Universiti Teknologi MARA, Bandar Puncak Alam, Selangor, 42300, Malaysia

##### **Correspondence:** Ahmad Fauzi Dali (fauzi.dali@uitm.edu.my)

Antimicrobial resistance (AMR) is defined as the ability of microorganisms to withstand the effects of antimicrobial agents. AMR has been recognised as one of the global health threats which can lead to anti-infective treatment failure. This will eventually increase patient’s medical costs and burden the health care system. Good understanding on AMR is important in reducing the emergence of new superbugs in the future. This study is aimed to assess the knowledge, attitudes and perceptions on antimicrobial use and resistance among pharmacy students in Universiti Teknologi MARA (UiTM) Selangor, Malaysia. A 25-item self-administered questionnaire was adapted from Dyar et al. (2013) and Rajiah et al. (2015). Survey tool content was validated by experts prior to pilot-testing. Questionnaires were distributed by hand and participant selection was done by stratified random sampling based on the inclusion and exclusion criteria. Data collection was done from March to May 2019. Statistical Package for Social Science (SPSS) version 25 was used for data analysis. Ethical approval was obtained from UiTM Research Ethics Committee. Two hundred and seven questionnaires were returned by the respondents which yielded a response rate of 100%. Based on sociodemographic characteristics, majority of the respondents were female (n=176, 85%), with current CGPA of 3.00 – 3.49 (n=135, 65.2%), in Year 4 of study (n=104, 50.2%) and living in urban residential area (n=121, 58.5%). All respondents (n=207, 100%) had high level of knowledge on AMR with a median score (IQR) of 10 (3). The median knowledge score of Year 4 students (median score (IQR) of 11 (2)) was significantly higher than of Year 1 (median score (IQR) of 8 (2)) (p<0.001). Year of study was significantly associated with the respondents’ level of knowledge (p<0.001) and perceptions (p<0.001). Attitude was found to be significantly associated with two sociodemographic factors namely residential area (p=0.034) and year of study (p<0.05). High knowledge, positive attitudes and good perceptions towards antimicrobial use and resistance were observed among pharmacy students in UiTM. To improve current understanding, attitudes and perceptions on AMR, continuing professional education among UiTM pharmacy students should be emphasised in the future.

## 26 Factors Affecting Modification of Initial Antiretroviral Therapy Regimen in HIV Positive Patients

### Min H. Cheah^1^, Eon T. Gan^2^, Nur J. Azman^3^, Najma Kori^4^, Petrick Periyasamy^4^, Siti-Azdiah Abdul-Aziz^1^

#### ^1^Faculty of Pharmacy, National University of Malaysia (UKM); ^2^Infectious Disease Clinic, Hospital Sultan Haji Ahmad Shah, Pahang, Malaysia; ^3^Pharmacy Department, Hospital Canselor Tuanku Muhriz, Kuala Lumpur, Malaysia; ^4^Infectious Disease Clinic, Hospital Canselor Tuanku Muhriz, Kuala Lumpur, Malaysia

##### **Correspondence:** Siti-Azdiah Abdul-Aziz (sitiazdiah@gmail.com)

Human immunodeficiency virus (HIV) infection is a disease that requires lifelong antiretroviral therapy (ART). It is essential to increase the durability of initial ART regimen, which subsequently improves clinical outcomes and preserves future ART options. The aim of this research is to investigate the reasons and factors associated with ART regimen modification among treatment-naïve HIV positive patients in infectious disease clinics of two tertiary hospitals in Malaysia. A retrospective observational study was conducted on all patients attending infectious disease clinic initiated on initial ART regimen between January 2013 to December 2017. All ART regimen modifications that occurred during the first year post ART initiation including related data were recorded. Binomial logistic regression was performed to analyze predictive factors for regimen modification. Among the 166 patients initiated on ART, 49 (29.5%) required ART regimen modification. A majority (69.4%) of the patients underwent regimen switch and the remaining had single drug substitution. Adverse drug reactions (ADRs) were the most commonly reported reasons for modification (79.6%), followed by poor adherence (8.2%). Poor adherence [adjusted odds ratio (OR) 4.233; 95% confidence interval (CI) 1.403-12.772; p = 0.010] and type of initial ART regimen were found to be independently affecting regimen modification. Regimens including zidovudine (AZT)/lamivudine (3TC)/efavirenz (EFV) (adjusted OR 3.880; 95% CI 1.647-9.143; p = 0.002), AZT/3TC/nevirapine (NVP) (adjusted OR 7.489; 95% CI 2.475-22.660; p < 0.001) and abacavir (ABC)/3TC/EFV (adjusted OR 8.714; 95% CI 1.241-61.166; p = 0.029) were associated with higher likelihood of modification compared to tenofovir (TDF)/ emtricitabine (FTC)/EFV. In conclusion, ADR was the main reason for initial ART regimen modification. Poor adherence resulted in higher rates of ART modification, TDF/FTC/EFV regimen was least associated with modifications compared to other first-line ART regimens recommended in Malaysia. Interventions to improve compliance and manage ADR effectively may prolong the duration of initial ART regimen and greatly benefit the patients.

## 27 The Use of Energy Medicines (EM), Manipulative Body Based Therapies (MBBT), Therapies from Whole Medical System (WMS) and Health Related Quality of Life (HRQoL) of thalassemia patients

### Wan Ismahanisa Ismail^1^, Mohamed Azmi Ahmad Hassali^2^, Maryam Farooqui^3^, Muhammad Nabil Fikri Roslan^1^Nurhidayah Ab.Rahim^1^, Syarifah Masyitah Habib Dzulkarnain^1^

#### 1Faculty of Health Sciences UniversitiTeknologi MARA, Penang, Malaysia; 2School of pharmaceutical sciences, UniversitiSains Malaysia, Penang, Malaysia; 3Department of Pharmacy Practice, Unaizah College of Pharmacy, Qassim University, Qassim, Saudi Arabia

Complementary and Alternative medicines (CAM) is common use among people with patients that difficult to treat or chronic illness such thalassemia patients. Therefore, this study to aims to determine the use of Energy Medicines (EM), Manipulative Body Based Therapies (MBBT), Therapies from Whole Medical System (WMS) and Health Related Quality of Life (HRQoL) among thalassemia patients. This cross sectional was performed among 390 thalassemia patients from Thalassemia Society Club, Kedah Malaysia using self-administrated questionnaire and HRQoL was assessed by using Short Form Health Survey with Only 36 Questions(SF-36). A total of 390 eligible thalassemia patients, 313 (80.26%) reported to have use CAM for their condition. A total of 202 (51.8%) reported to use different types of EM, MBBT and WMS therapies. The majority of the EM, MBBT and WMS users were female 130 (64.4%), aged between 18 to 27 years 123 (60.96%) and were Malay ethnicity 193(95.5%) and were muslims 187(92.6%, p<0.05). Therapies from WMS such as traditional Malay medicines 126(62.4%), traditional Chinese medicines 20(9.9%) and homeopathy 47(23.3%) were commonly used by participants. Only 44 (14.1%) reported to spend more than 100 Ringgit Malaysia(MYR) and 208 participants could not estimate their monthly expenditure for CAM treatment. Majority type of β-thalassemia patients were most use 93(46.0%). No significant different found in SF-36 HRQoL score between EM,MBBT and WMS (p>0.05). The use of WMS was common use among thalassemia patients. Further research and heath education is required to evaluate safe and practice of these therapies in thalassemia.

## 28 Therapeutic interchange policies used in managing antimicrobial shortages in South African public sector hospitals

### Audrey K Chigome,Moliehi Matlala, Johanna C Meyer

#### Department of Public Health Pharmacy and Management, School of Pharmacy, Sefako Makgatho Health Sciences University, South Africa.

Antimicrobial shortages are regarded as a public health crisis due to the necessity to expedite treatment of infection and because antimicrobial resistance limits therapeutic choices. Therapeutic interchange policies have been documented elsewhere are useful in dealing with antimicrobial shortages. The extent of antimicrobial shortages and availability of documented therapeutic interchange policies is largely unknown in South Africa. The objectives of this study was to describe the pharmacist’s practices and role in the therapeutic interchange process when there are antimicrobial shortages at public sector hospitals in South Africa. A quantitative and descriptive study design using an electronic questionnaire was administered via SurveyMonkey^TM^. The target population included 405 public sector hospitals across South Africa. Information on antimicrobial shortages, availability of documented therapeutic interchange policies was collected from pharmacists. Data were exported to MS Excel™ and analysed using SPSS™ v24. Descriptive statistics were used to summarise categorical variables as frequencies and percentages. Data collection is currently in progress. Most (83.3%) hospitals had experienced shortages in the previous six months. Antimicrobials commonly reported as out of stock included cloxacillin (54.3%), benzathine benzylpenicillin (54.2%), erythromycin (39.6%) and ceftriaxone (38.0%). Reasons for shortages included pharmaceutical companies with supply constraints (85.3%) and an inefficient supply system. Only 42.4% had therapeutic interchange policies, and 88.9% contacted the prescriber when there was a need for substitution. Antimicrobial shortages are prevalent in South African public sector hospitals with penicillins and cephalosporins being the most affected. Therapeutic interchange policies are not available at most hospitals. Effective strategies are required to improve communication between pharmacists and prescribers.

## 29 Practice of self-medication among students in a public university

### Nur Hazirah Izzati Zaidi, Kamaliah Md Saman, Mathumalar Loganathan Fahrni

#### Department of Pharmacy Practice, Faculty of Pharmacy, Universiti Teknologi MARA Cawangan Selangor Kampus Puncak Alam, 42300 Bandar Puncak Alam, Selangor, Malaysia

##### **Correspondence:** Kamaliah Md Saman (kamaliah@uitm.edu.my)

Self-medication is a global phenomenon and while it can be convenient to patients, it can potentially lead to increased side-effects and adverse reactions as well as exacerbate current health conditions, when misused for a prolonged period. This study aimed at determining knowledge, prevalence and factors influencing self-medication, sources of supply, the types of medication involved and the association between demographic data and knowledge, attitude and practice of self-medication among students of a public university. A cross-sectional study using convenience sampling was conducted among students from various faculties of Universiti Teknologi MARA at the Puncak Alam Campus. Questionnaires were self-administered. Analysis was done using SPSS version 24. Out of 370 questionnaires distributed, a total of 354 questionnaires were returned. The majority of the respondents were aged between 19 to 22 years old (64.7%), were undergraduate pharmacy students (31.9%) and were predominantly female (83.6%). The prevalence of self-medication was 59.9%. Approximately one-third (31.4%) of the respondents were able to define self-medication correctly. The primary factors that motivated students to self-medicate were that they did not have to visit the doctor for minor illnesses (25.5%), it saved them considerable time (23.3%) and that it offered them quick-relief (15.2%). Analgesics (22.4%), multivitamins (21.5%) and antipyretics (12.9%) were the most common classes of medications used for self-medicating. There were significant associations between respondents’ age and attitude as well as practice. There was a significant association between gender and education with practice. There were also significant associations between faculties students were attached to with their knowledge, attitude and practice of self-medication. More than half of the respondents (53.4%) stated that self-medication should not be encouraged given its potential for misuse. Pharmacies were highly sourced for medications (51.5%). Nevertheless, the prevalence of self-medication was found to be low compared to previous studies. The respondents showed a positive attitude towards self-medication. Nevertheless, as two-thirds were unable to define self-medication accurately, this suggested that students may have underestimated the harms and risks that were associated with the practice of self-medication. We recommend that safe and effective self-practice modules are to be incorporated in the curriculum of students.

## 30 A Qualitative Study: Exploring Pharmacist-Physician Collaborative Practice In DMTAC At Primary Care Health Clinic

### Fajaratunur A.Sani^1,2^ , Shubashini Gnanasan^2^ ,Mahmathi Karuppannan^2^

#### ^1^Muar District Health Clinic, Johor; ^2^Faculty of Pharmacy, Department Of Pharmacy Practice, University Technology MARA(UiTM),Malaysia

##### **Correspondence:** Fajaratunur A. Sani (anursha2001@yahoo.com)

Diabetes Medication Therapy Adherence Clinic (DMTAC) is ambulatory care service which was introduced in Malaysia in 2004. This service involves collaboration between pharmacists and physicians in providing medication therapy management to diabetic patients. Collaborations between health-care providers is a relatively new concept in the field of pharmacy, particularly in primary care health clinics. Effective collaborative practices are highly dependent on the good relationship and teamwork between professional health-care team members especially pharmacists and physicians. The objectives are to explore the perception of pharmacists and physicians on the contributing factors and challenges in the implementation of DMTAC at the primary care health clinics. A qualitative approach of four focus groups consisting of pharmacists and physicians were conducted at four different health clinics in Muar district between February 2015 to March 2015. A semi-structured interview questionnaire was used to facilitate discussions. Conversations were audio-recorded, transcribed verbatim and coded by themes. This study was approved by the Medical Research and Ethics Committee, Ministry of Health, Malaysia, Faculty of Pharmacy Research Ethics Committee and UiTM Research Ethics Committee. Twelve pharmacists and ten physicians with the average of six years of service participated in the study. The physicians and pharmacists emphasized on few factors contributed to the effective collaboration between them such as giving feedbacks, being aware and respectful of each other’s roles, conducive working environment and should have adequate number of staffs. Existing DMTAC protocols and Diabetes Clinical Practice Guidelines (DCPG) should be implemented especially in the flow of processes to make them more practical and adaptable to be used locally. This study revealed the expectations of physicians with regards to the collaborative practices of which pharmacists should be more open and willing to share and communicate effectively their findings on medication related problem in improving patient care. Pharmacists in the government health clinics may have limitations in servicing the public, but they have the capability to cultivate and implement drug therapy management through MTAC in managing Type 2 diabetes patients. Effective communication and collaboration between pharmacists and physicians may result in better care for patients with diabetes at the health clinics.

## 31 Assessment and Implementation of Proper Use of MDI among Unaizah Pharmacy and Medical Female Students

### Dema Nasser Alhebs

#### Unizah College of Pharmacy, Qassim University

Improper use of Metered dose inhaler (MDI) almost lead to failure of management of respiratory disorders and accompanied by respiratory complications, increasing cost of therapy, aggravating patient quality of life by growing incidence of mortality and morbidity. Lack of adequate instructions to patients about the proper way of using MDIs by HCPs. The ambiguous fact is that most of them are unaware of their role or don't know how to use MDIs properly. The objectives of this study were to assess the knowledge of UCP and UCM students regarding the role of MDI in the treatment of asthma and correct technique of using MDIs and the effect of a single educational intervention in improving their practical skills in using MDI. Cross-sectional, single interventional study included 157 female students of UCP and UCM students' of 3rd, 4th and 5th-year. They filled 10 MCQ questionnaire then they evaluated before and after one to one educational session about the proper use of MDIs using a validated evaluation form. Out of the 293 students from UCM, UCP only 147(41 UCM, 106 UCP) students were included in our study. Most students chose the responsibility of pharmacist and physician in educating patients, fewer choices for nurses. Only 46 (31%) students (27 UCM, 19 UCP) could use the MDI properly before education step this number was improved to 134(39 UCM, 95 UCP) in the post-education step while 13 made one or two mistakes. The Post-education number of students who did each step correctly was significantly improved (P-value< 0.5) except in step 3 and 5. UCP students who said they knew how to use MDI correctly was 60 while the actual number who knew was 19 which was significantly different (P-value=0.384) while it non significantly differed among UCM students ((P-value=0.049). Pharmacy students are in need for a robust assessment tool using a validated technique to ensure their ability to use MDI properly. one-time education intervention isn’t enough to train the students and to achieve excellent handling performance.

## 32 Behavioral and Psychological Symptoms through the Eyes of People with Dementia (PWD)

### Nur Sabiha Md Hussin, Shubashini Gnanasan, Mahmathi Karupannan, Yogheswaran Gopalan

#### Department of Pharmacy Practice, University Teknologi MARA, Bandar Puncak Alam, Selangor, Malaysia

Behavioral and Psychological Symptoms of Dementia (BPSD) are considered as spectrum of dementia. The need-driven, dementia-compromised behaviour (NDB) is a concept explaining BPSD as the result of combination of inability of the care providers to understand patient’s needs and the inability of the patient to express them. The symptoms may be due to unmet needs which is expressed by physical expression or emotional distress by PWD. Thus this study aims to understand the emotion, perception, action, experiences and the needs of PWD. Qualitative study design was employed. Data were collected through observations and semi-structured interviews with people with dementia PWD at seven nursing care centres. Observations were written in the field notes and interviews were audio recorded and then transcribed verbatim. All data were subjected to thematic analysis. The findings revealed the PWD favour engagement in physical activities be it indoor or outdoor activities such as traveling, dancing and playing musical instruments. They also wished for cognitive-oriented activities which include learning new language, spelling, colouring, reading and writing. PWD were also keen for social interaction involving human to human and human to animal communication. The collection of findings revealed their voices were less heard which has resulted in decreased satisfaction among PWD. The unmet needs can be categorised as need for environmental adjustment, social interaction and other undelivered non-pharmacological psychotherapeutic approaches. Therefore, continuous education and training for the healthcare providers are essential for them to recognise the key inducers of need-driven behavioural and psychological symptoms. Application of systemic algorithms and methodologies is also required to control the emergence of need-driven behavioural and psychological symptoms. Although there are ample strategies to cater the behaviour challenges among PWD, involvement of PWD in the care management by listening to their perspective may result in a more holistic management of need-driven behavioural and psychological symptoms

## 33 DRUG DOSAGE ADJUSTMENT IN THE TREATMENT DECISION MAKING

### Badamkhand Gankhulug,^2^ Otgonbileg Tegshee^2^, Gereltuya Dorj^1^, Tsetsegmaa Sanjjav ^1,3^ Bruce Sunderland,^4^ Gantuya Dorj^5^

#### ^1^School of Pharmacy, Mongolian National University of Medical Sciences, S. Zorig street, 14210 Ulaanbaatar, Mongolia; ^2^The Third Central Hospital of Mongolia, Bayangol district, 10^th^ khoroolol-2, Ard Ayush Avenue; ^3^Drug Research Institute, Monos group, Sonsgolon Road-5, Songinokhairkhan district, Ulaanbaatar 18130, Mongolia; ^4^Faculty of Health Sciences, Curtin University, Kent Street, Bentley, Perth, Western Australia 6102; ^5^School of Public Health, Mongolian National University of Medical Sciences, S. Zorig street, 14210 Ulaanbaatar, Mongolia

##### **Correspondence:** Badamkhand Gankhulug (g_badka@yahoo.com)

Drug dosage adjustment of certain medicines is required in patients with reduced renal function to avoid toxicity, because many of these medicines are eliminated by the kidneys. This study aimed to identify the appropriateness of treatment provided for inpatients, to assess the use of antimicrobial drugs and to establish the prevalence of patients requiring drug dosage adjustment. A retrospective cross-sectional study was carried out in the nephrology departments of tertiary level hospitals in Mongolia. Inpatients were randomly selected and information regarding the patient’s condition and medication were collected and analyzed. Estimation of creatinine clearance was completed using the Dottley and the Gaul-Cockroft methods. Categorical differences were tested by means of χ2 and Fischer test. A total of 100 patients admitted to the nephrology wards were collected, of which 57% were male (n = 57), the mean age was 46 years and most of participants were aged between 66-80 years old (30.1%). The mean hospitalization duration was 11.4 days (5-25 days). A total of 839 medicines were prescribed. The average number of prescribed drugs was 1.9 per patient. Of all prescribed medicines, 76.8% (n = 653) were injections, 23.2% were oral and other forms. At least one antibiotic was prescribed all patients. Twenty-six patients required drug dosing adjustment. Drug dosing was not adjusted with significantly more elderly patients with SrCr <50 ml/min were observed (*p* = .0001). Our study suggested that about 1/3 of all hospitalized patients required drug dosing adjustment. The pharmacotherapy administered for patients with renal impairment should be controlled and monitored to improve the quality of care provided in Mongolian hospitals.

## 34 A Compatibility Study of Injection Drugs Marketed in Mongolia

### Azjargal Ganbat,^1, 2^ Gereltuya Dorj, ^1^ Tsetsegmaa Sanjjav ^1,3^ Bruce Sunderland,^4^ Gantuya Dorj^5^

#### ^1^School of Pharmacy, Mongolian National University of Medical Sciences, S. Zorig street, 14210 Ulaanbaatar, Mongolia; ^2^Mongolian University of Pharmaceutical Sciences, Sonsgolon Road-^5^,Songinokhairkhan district, Ulaanbaatar 18130, Mongolia; ^3^Drug Research Institute, Monos group, Sonsgolon Road-5, Songinokhairkhan district, Ulaanbaatar 18130, Mongolia; ^4^Faculty of Health Sciences, Curtin University, Kent Street, Bentley, Perth, Western Australia 6102; ^5^School of Public Health, Mongolian National University of Medical Sciences, S. Zorig street, 14210 Ulaanbaatar, Mongolia

##### **Correspondence:** Azjargal Ganbat (azjargal.g@monos.mn)

In case for gram-negative severe infections like osteomyelitis and sepsis, ciprofloxacin injection can be used in combination with other antibiotics such as cefuroxime and cefotaxime. In clinical practice, the research of chemical and physical compatibility of injections are rarely found. Therefore, the current study aimed to identify the compatibility of ciprofloxacin injection with other parenteral antibiotics marketed in Mongolia. The study purpose is to determine the compatibility of ciprofloxacin, to complete a post marketing quality study of a commonly used drug in Mongolia. Ciprofloxacin injection and cefuroxime sodium tablets were selected in this study. The compatibility research was completed according to the methodology published by the Elmore et al. Quantitative analysis was completed by means of HPLC. Ciprofloxacin parenteral solution was mixed with cefuroxime, the concentration had dropped by 0.2 units after 6 and 24 hours, respectively. After testing the state of ciprofloxacin and cefuroxime mixed sodium solution at certain time periods, precipitation in the ciprofloxacin and cefuroxime mixed sodium solution was observed after 6 hours. The study has shown that the pH of mixed solution has decreased, as well as the concentration of ciprofloxacin has decreased along with time, visual testing revealed that precipitation was developed within 6 and 24 hours.

## 35 PHARMACY TECHNICIAN’S PROFESSIONAL COMPETENCY AND COMPETENCY-BASED CURRICULUM DEVELOPMENT

### D.Ariunaa, S. Purevsuren , S.Tugsbileg, B.Boditsetseg, D.Baigalmaa, B.Bolor, B.Otgonbat, P.Mandahnaran

#### Department of Pharmacy Technician, School of Pharmacy, Mongolian National University of Medical Sciences , 14210 Ulaanbaatar city, Sukhbaatar district, S. Zorig Street,, Mongolia )

##### **Correspondence:** D.Ariunaa (ariunaa.ds@mnums.edu.mn)

**Keywords:** Pharmacy technician, Professional competency, Competency-based Curriculum

According to the international trends, а competency-based curriculum is dominating in vocational and technological (college) education. It is significant to define the competence of pharmacy technicians for developing the curriculum.

In Mongolia, the pharmacy service was focused on the providing the medications; nevertheless now this condition is gradually shifting to patient or client-centered service. Consequently, we see the need in preparing a pharmacy technician with globally required competencies (team-working, ethic-based and etc.) Our research goal was to determine and define developing the pharmacy technician competencies to develop the curriculum. We emplyoed the qualitative research methods for interviewing, summarizing and compiling based on the document materials referring to the professional competencies of Singapore and Canada. According to the documents that approved by Health Ministry of Singapore (“National Competency Standards for Pharmacy Technicians (Entry-Level) 2015”) and Canadian National Association of Pharmacy Regulation/ Association of Faculties of Pharmacy of Canada (AFPC)/ (“Professional Competencies for Canadian Pharmacy Technicians at Entry to Practice, 2014”) pharmacists should acquire the following 9 competencies: professional ethics, judiciary and responsibilities, personal and interpersonal communication knowledge and skills, professional basic knowledge and skills, preparation of sterile and non-sterile compounding, receipt, trade, store and distribute the products, patient care, technology and info system, pharmacy regulation, provide drug quality and safety. It is evident that the above-mentioned competencies could close to our mission. And it could be served us as the role model of a good curriculum.

## 36 A Cross Sectional Assessment of Complementary and Alternative Medicines (CAM) use among Chronic Kidney disease (CKD) patients, in Unaizah, Qassim

### Asma Alhatlani^1^, Nujud Alshehaitan^1^, Amjad Alherabi^1^, Dr.Maryam Farooqui^2^

#### ^1^Uniazah College of Pharmacy, Qassim University, Saudi Arabia; ^2^Department of Pharmacy Practice, Unaizah College of Pharmacy , Qassim University , Saudi Arabia

Despite the paucity of scientific evidence, CAM is widely used for the prevention and treatment of illness among patients with chronic diseases including CKD. It is evident that the irrational use of CAM among CKD may affect renal function and may lead to renal failure. General aim is to investigate the CAM use among CKD patients and to compare CAM practice with different demographic and disease characteristics of the participants. The specific objectives are to determine the prevalence of CAM use among CKD patients, to explore the most common CAM used by CKD patients, to identify the monthly expenditure of CAM among CKD patient, to evaluate how far CKD patients, disclose their CAM use to their health care providers. This quantitative study was conducted from December 1, 2018 to February 1, 2019 in nephrology wards in King Saud Hospital and the hemodialysis center (Daiverum). Face-to-face questionnaire-based interviews were held with 170 CKD patients out of 191 (response rate, 89%). The completed questionnaires were computed in SPSS for data analysis. Descriptive and inferential analysis was use to present the data. A total of 170 patients completed the questionnaire among those 60 (35.3%) were the CAM users. A wide range of CAM were used by the CAM users among which the most common were herbal products 49 (81.6%) and spiritual therapies 34 (56.6%). Nearly half 47 (78.3%) CAM users reported to spend <50 Saudi Riyal for CAM. The friends and family members 28 (46.6%) were the most common source to get information on CAM followed by social media and internet 21 (35%). Among CAM users 27 (45%) reported to disclose CAM use to their health care providers. The most common reason of non-disclosure was it is not important for the doctors know about their CAM use 16 (50%). Among the CAM users who discontinue their using of CAM 28 (46.6%), when asked the reasons of not using CAM, the reason given was no benefit of CAM in CKD 20 (71.4%). Among all the demographic and disease characters only gender was significantly associated with CAM used (p=0.015). In conclusion, the use of CAM among CKD patients was relatively high. Although the safety and efficacy of herbal preparations in CKD is not established however majority of the participants reported to use herbal preparations which raise concerns towards the unknown effects of CAM on renal function. Patients reported to use CAM without disclosing to their health care providers which is a major health risk. It is critical to counsel CKD patients regarding rational and informed CAM use in order to prevent unwanted effects on renal function.

## 37 Adopting Mission Command to Improve Waiting Time in Outpatient Pharmacy Department of 95 Tuanku Mizan Armed Forces Hospital

### Gill M.S.^1^, Basari AH^2^, Adnan MA^3^, Mat Rahim MA^4^

#### ^1, 4^ Dept of Pharmacy, 95 Armed Forces Hospital, Ministry of Defense, Malaysia; ^2^ Health Services Division, MAF HQ, Ministry of Defense, Malaysia; ^3, 5^ 93 Armed Forces Medical & Dental Depot, Ministry of Defense, Malaysia.

Long waiting hours in Outpatient Pharmacy Department (OPD) has been a major problem. For Q1 and Q2 of 2018, OPD filled 15,473 and 14,302 prescriptions. The average monthly patient waiting times were 35 to 45 minutes, exceeding 30 minute target. Patient satisfaction study shows 57.8% of responders branded the waiting time as satisfying but requested for improvement. This report analyses the impact of using mission command principle to achieve positive working output in OPD. The indicator measured is the monthly average patient waiting time after intervention introduced in September 2018. For Q3 and Q4 of 2018, OPD filled 16,385 and 16,696 prescriptions. Average waiting times in September until December 2018 are respectively 29:05, 27:35, 21:11 and 15:07. Patients receiving medications within 30 minutes has increased to 100% for Q4 of 2018 and Q1 of 2019. Military operations demand continuous and mutual adaptation among all participants. Health service support in operations conducts in complex, ever-changing and uncertain environments. Thus, mission command philosophy was introduced at OPD tactical level. This was guided by the principles of building cohesive teams through mutual trust, create a shared understanding, provide clear intent, exercise disciplined initiatives, use mission orders and accept prudent risk. The principles of mission command assist commanders and staff in blending the art of command with the science of control. Together, the mission command philosophy and OPD objective quality will guide, integrate and synchronize the pharmacy team throughout the conduct of unified operations.

## 38 Diabetes self-care knowledge: A cross-sectional survey among patients with type 2 diabetes mellitus in Hospital Sultanah Bahiyah, Alor Setar

### Nurul Atika Mohd Fauzi^1^, Nurin Hasya Hamidi^1^, Muhammad Amin Azizul Rahman^1^, Norazila Abdul Ghani^2^, Qi Ying Lean^1,3^, Chin Fen Neoh^4,5^,Yuet Yen Wong^1^

#### ^1^Faculty of Pharmacy, Universiti Teknologi MARA, Cawangan Pulau Pinang, Kampus Bertam, Penang, Malaysia; ^2^Pharmacy Department, Hospital Sultanah Bahiyah, Alor Setar, Malaysia; ^3^Vector-Borne Diseases Research Group (VERDI), Pharmaceutical and Life Sciences CoRe, Universiti Teknologi MARA, Shah Alam, Selangor, Malaysia; ^4^Faculty of Pharmacy, Universiti Teknologi MARA, Cawangan Selangor, Kampus Puncak Alam, Selangor, Malaysia; ^5^Collaborative Drug Discovery Research (CDDR) Group, Pharmaceutical and Life Sciences Community of Research, Universiti Teknologi MARA, Shah Alam, Selangor, Malaysia

##### **Correspondence:** Yuet Yen Wong (yuetyen@yahoo.com)

Type 2 diabetes mellitus (T2DM) affects 1 in 5 Malaysians age more than 30-year-old. Despite a plethora of hypoglycaemic agents, glycaemic control remains poor among substantial T2DM patients. Diabetes self-care warrants in-depth exploration as it plays integral role in achieving good glycaemic control. This study therefore aimed to (1) assess self-care knowledge among T2DM patients attending Hospital Sultanah Bahiyah (HSB); (2) explore factors affecting patient’s diabetes self-care knowledge; (3) determine the relationship between diabetes self-care knowledge and glycaemic control (i.e. fasting blood glucose and HbA1c). A cross-sectional survey, using pre-validated questionnaire, was conducted over 3 months (Dec 2018 - February 2019) at outpatient pharmacy department (OPD) in HSB. Convenience sampling method was used. Patients who fulfilled inclusion and exclusion criteria were consented to the study. The total diabetes self-care knowledge score was calculated based on the correct responses provided. The score was then converted into percentage with more than 70% deemed to have good knowledge. All data was analysed using IBM statistical package for social sciences (SPSS) version 20 with a priori p < 0.05 considered statistically significant. A total of 240 questionnaires were completed and returned. The median total diabetes self-care knowledge score was 21 [Interquartile range (IQR) 7] with score range of 10 to 30. More than half of respondents (55%) scored high knowledge (i.e. > 70%) with several knowledge gaps remained particularly about drug regimen. Only 13.8% of respondents knew the function of aspirin and less than half (45.8%) aware that alcohol could interact with diabetic drugs. Our findings showed that respondents’ diabetes self-care knowledge scores were significantly different with the level of education (p = 0.003) and counselling received (p = 0.013). No significant differences in the knowledge scores were observed in other sociodemographic factors (i.e. age, gender, ethnic group, monthly household income and duration of T2DM diagnosis). We observed significant weak negative correlation between diabetes self-care knowledge scores and HbA1c (r = - 0.015, p < 0.001). Similarly, a significant medium negative correlation was noted between knowledge scores and fasting blood glucose (r = - 0.397, p < 0.001). More than half of T2DM patients (55%) in HSB have high diabetes self-care knowledge level but several knowledge gaps remained. Better diabetes self-care knowledge was noted among patients with higher educational level and had received counselling. Healthcare professionals need to address patient’s diabetes self-care knowledge gaps because higher self-care knowledge is correlated with better glycaemic control.

## 39 Self-care behaviours among hypertensive patients in Hospital Sultanah Bahiyah, Alor Setar

### Izzah Zahra Nasrudin^1^, Nur Izzati Mohd Zaki^1^, Muhammad Fareez Nazwaad Ismail^1^, Norazila Abdul Ghani^2^, Qi Ying Lean^1,3^, Chin Fen Neoh^4,5^, Yuet Yen Wong^1^

#### ^1^Faculty of Pharmacy, Universiti Teknologi MARA, Cawangan Pulau Pinang, Kampus Bertam, Penang, Malaysia; ^2^Pharmacy Department, Hospital Sultanah Bahiyah, Alor Setar, Malaysia; ^3^Vector-Borne Diseases Research Group (VERDI), Pharmaceutical and Life Sciences CoRe, Universiti Teknologi MARA, Shah Alam, Selangor, Malaysia; ^4^Faculty of Pharmacy, Universiti Teknologi MARA, Cawangan Selangor, Kampus Puncak Alam, Selangor, Malaysia; ^5^Collaborative Drug Discovery Research (CDDR) Group, Pharmaceutical and Life Sciences Community of Research, Universiti Teknologi MARA, Shah Alam, Selangor, Malaysia

##### **Correspondence:** Yuet Yen Wong (yuetyen@yahoo.com)

In Malaysia, hypertension is reported to affect 1 in 3 adults age 18-year-old and above. Among those who have their hypertension treated, only one third could achieve good blood pressure control. Considering the high prevalence but poor disease control among hypertensive patients in Malaysia, it is urgently needed to assess current self-care behaviour given that hypertension is largely self-managed by patients outside of the clinic setting. This study therefore aimed to (1) assess self-care behaviour among hypertensive patients in Hospital Sultanah Bahiyah (HSB); (2) explore factors contributing to hypertension self-care behaviour; (3) determine relationship between hypertension self-care behaviour and blood pressure control. A cross-sectional survey, using pre-validated questionnaire, was conducted over 4 months (Nov 2018 - February 2019) at outpatient pharmacy department (OPD) in HSB. Convenience sampling method was used. Patients who fulfilled study inclusion and exclusion criteria were consented to the study. The total hypertension self-care score was calculated based on a 4-point Likert scale (1 = rarely; 2 = sometimes; 3 = often; 4 = always) regarding the likelihood of patients performing each self-care behaviour). All data was analysed using IBM statistical package for social sciences (SPSS) version 20 with a priori p < 0.05 considered statistically significant. A total of 160 questionnaires were completed and returned. The median total scores for hypertension self-care behaviour was 46 [Interquartile range (IQR) 14] with minimum scores of 20 and maximum scores of 80. Our findings showed that the respondents’ self-care behaviour scores were significantly different with ethnicity (p = 0.015), level of education (p < 0.001), occupation (p < 0.001) and monthly household income (p < 0.001). No significant differences in self-care behaviour scores were observed in other sociodemographic factors (i.e. gender and living status). Furthermore, self-care behaviour scores were significantly negative correlated with age (r = -0.409, p < 0.001) and duration of having hypertension (r = - 0.289, p < 0.001). The data suggested that hypertensive patients with older age and longer duration of hypertension tend to have poorer self-care behaviour scores. We observed a significant weak negative correlation between self-care behaviour scores and systolic blood pressure readings (r = - 0.196, p = 0.013). Self-care behaviours among hypertensive patients in HSB remained sub-optimal. It is also essential for healthcare professionals to devise appropriate intervention in engaging hypertension self-care.

## 40 Public Awareness and Practices Towards Self-Medication with Antibiotics Among Malaysian Population: Questionnaire Development and Pilot Testing

### Adeel Aslam^1^, Che Suraya Zin^1^, Norny Syafinaz^1^, Syed Imran Ahmad^2^, Shazia Jamshed^1^

#### ^1^ Pharmacy Practice Department, Kulliyyah of Pharmacy, International Islamic University Malaysia, Kuantan, Pahang, Malaysia; ^2^ Pharmacy Practice Department, School of Pharmacy, International Medical University, Malaysia

##### **Correspondence:** Shazia Jamshed (shazia_jamshed@iium.edu.my)

Self-medication with antibiotics (SMA) is a common global feature due to knowledge, choices, and alternatives when taking care of own health. However, this inappropriate practice generally leads towards antibiotic resistance. The aim of this study was to validate and develop an instrument in Bahasa Melayu to assess the awareness and to evaluate practices towards SMA among the Malaysian population. A pilot study was conducted among the 100 Malaysian participants through translated (Bahasa Melayu version) research instrument. Reliability testing, (test-retest, internal consistency, content validity) was applied to establish the reliability. In addition, descriptive statistics applied along with other tests such as: one-way ANOVA, t-test and Pearson correlation were applied to determine significant differences between groups. A panel of nine experts evaluate the research instrument for content validity and results demonstrate that this research instrument had strong content item validity (Indices = 1). Each domain (level of knowledge and understanding about antibiotic use and antibiotic resistance: Practice towards self-medication: Patient-reported outcomes) showed good internal consistency of Cronbach’s alpha 0.658, 0.90 and 0.670 respectively. While test-retest reliability value for each domain were 0.773 (p = 0.009), 0.891 ((p = 0.001), and 0.787 (p = 0.007). The mean ± standard deviation (SD) for the level of knowledge about antibiotic use was 3.35 ± 1.89 and level of knowledge and understanding about antibiotic resistance 4.11 ± 1.82. The most common reason for SMA was lack of trust towards doctors 52% and prevalence towards SMA was 19%. Based on these analyses, this instrument demonstrated reliability and validity and therefore is considered an effective tool to assess public awareness and practices towards self-medication with antibiotics among the Malaysian population.

## 41 A study on the adulterated traditional-herbal medicinal products in Malaysia

### Authors: H. Yahaya^1^, H. A. Suriana^2^, A.W. Izyan^3^, R. Amlizan^4^

#### ^1^Management and Science University; ^2^Pharmacy Enforcement Division, Ministry of Health Malaysia; ^3^Cyberjaya University College of Medical Sciences; ^4^MARA University of Technology.

The aims of this study are to identify characteristics of seized, adulterated Traditional-Herbal Medicine (THM) products in Malaysia and to assess Adverse Reactions (ARs) signals associated with THM products. A total of 59, 440 THM products that were seized by the Malaysia Pharmacy Enforcement Division between 2008–2014 were analysed. Of these, 6, 452 THM products were included in the final analyses after taking into consideration the inclusion and exclusion criteria. To quantify the THM ARs signals, ARs reports for THM products were obtained from the National Pharmaceutical Regulatory Agency Adverse Drug Reaction Database. Proportional Reporting Ratio (PRR) and Reporting Odds Ratio (ROR) are safety signalling tools that were used to measure THM ARs signals. More than 50% (n=3, 549/6, 452) of the adulterated THM products originated from international countries where the highest was from Indonesia (45.68%, n=2, 947/3, 549). The most common adulterated THM product were claimed for pain and fever (53.64%, n=3, 461/6, 452), followed by sex stimulant (26.38%, n=1, 702/6, 452), and slimming (8.2%, n=529/6, 452). Steroids showed as the highest adulterant found in the adulterated THM products (47.78%, n=3, 083/ 6, 452), followed by phosphodiesterase inhibitor (25.77%, n=1, 663/6, 452), monoamine reuptake inhibitor (8.23%, n=531/6, 452), and non-steroidal anti-inflammatory drugs (NSAIDS) (5.64%, n=364/6, 452). A total of 1, 102 ARs reports for THM products were extracted but only 900 AR reports were eligible to be analysed using PRR and ROR. Hepatobiliary disorders (n=155, 17.22%) was the most common ARs with the use of THM products in Malaysia, followed by blood and lymphatic disorders (N=149, 16.56%) and gastrointestinal disorders (n=68, 7.56%). AR signals were generated for: 1) respiratory & thoracic disorders and THM products that are claimed for cough and cold, 2) cardiac disorders for THM products that are claimed for slimming and 3) reproductive and breast disorders for THM products that are claimed for women’s health. The results of this study highlight the trend of adulterated THM products seized in Malaysia and the ARs associated with the use of THM products. Strategic and systematic actions can be planned and implemented by the health authorities targeting the common adulterated THM products. A targeted awareness and educational programmes about THM products can be effectively undertaken for the public as well.

## 42 Impact of consultation with infectious disease specialists on inpatient mortality attributable to nosocomial infections

### Tuangrat Phodha^1,2^, Arthorn Riewpaiboon^2^, Kumthorn Malathum^3^, Peter C Coyte^4^

#### ^1^ Center of Excellence in Pharmacy Practice and Management Research, Drug Information and Consumer Protection Center, Faculty of Pharmacy, Thammasat University, Pathumthani, Thailand; ^2^ Faculty of Pharmacy, Mahidol University (Graduate Program), Bangkok, Thailand; ^3^ Division of Infectious Disease, Department of Medicine, Faculty of Medicine Ramathibodi Hospital, Mahidol University, Bangkok, Thailand; ^4^ Institute of Health Policy Management and Evaluation, School of Public Health University of Toronto, Ontario, Canada

##### **Correspondence:** Kumthorn Malathum (kumthornid@gmail.com), (mkumthorn@yahoo.com)

Infectious disease is a major health threat worldwide, particularly in countries with limited resources where the prevalence of infections is high but the number of specialists is inadequate. This study estimates the impact of consultation with infectious disease specialists (IDS) on the inpatient mortality rate of nosocomial infection caused by multidrug-resistant organisms. A prospective cohort study was conducted at Ramathibodi Hospital, Bangkok, a 1,400-bed university hospital in Thailand, between 1^st^ November 2013 and 30^th^ June 2014. A multivariate Cox proportional hazard regression model was used to estimate the impact on inpatient mortality related to consultation with IDS after controlling for a number of potential confounders including a propensity score (PS). Covariates included in the Cox model comprised of PS, consultation with an IDS, time to consultation with an IDS, sex, admitted ward, Charlson co-morbidity index score, site of infection, number of nosocomial infection episode, susceptibility group, and type of bacteria. Consultation with IDS improved inpatient survival by 44% while holding all other variables constant. Earlier consultation with infectious disease specialists by one day would yield an absolute improvement in inpatient mortality of 0.26%. Consultation with IDS was very important as it improved the rate of inpatient survival. IDS consultation should be performed early in the course of nosocomial infection to obtain a better survival outcome for these patients.

## 43 Intrapersonal factors associated with disease self-management among adolescent and youth patients with type 2 diabetes mellitus (T2DM): a meta-synthesis

### Nursyuhadah Othman^1,2^, Yuet Yen Wong^2^, Qi Ying Lean^2,3^, Nurain Mohd Noor^4^, Chin Fen Neoh^1,5^,

#### ^1^Faculty of Pharmacy, Universiti Teknologi MARA (UiTM), Cawangan Selangor, Kampus Puncak Alam, 42300 Bandar Puncak Alam, Selangor Darul Ehsan, Malaysia; ^2^Faculty of Pharmacy, Universiti Teknologi MARA (UiTM), Cawangan Pulau Pinang, Kampus Bertam, 13200 Kepala Batas, Pulau Pinang, Malaysia; ^3^Vector-Borne Diseases Research Group (VERDI), Pharmaceutical and Life Sciences CoRe, Universiti Teknologi MARA, Shah Alam, Selangor, Malaysia; ^4^Endocrine Unit, Hospital Putrajaya, Precint 7, 62250, Putrajaya, Malaysia; ^5^Collaborative Drug Discovery Research (CDDR) Group, Pharmaceutical and Life Sciences Community of Research, Universiti Teknologi MARA (UiTM), 40450 Shah Alam, Selangor Darul Ehsan, Malaysia; ^6^Clinical Research Centre, Hospital Putrajaya, Precint 7, 62250, Putrajaya, Malaysia.

##### **Correspondence:** Chin Fen Neoh (neohchinfen@puncakalam.uitm.edu.my)

Type 2 diabetes mellitus (T2DM), once thought adult-onset is now affecting the adolescents and youth due to parrellel increase of obesity and overweight resulting from sedentary lifestyle and unhealthy diet. T2DM is associated with increased risk of cardiovascular diseases and mortality; however, such complications can be mitigated by good glycaemic control. Patient-driven self-management is the cornerstone of T2DM management. While the extent and capability of disease self-management among adolescent and youth remain largely unknown, this population may have limited abilities to make own judgement in daily self-management outside of the clinic setting. The aim of this study is to identify the intrapersonal factors which influenced self-management behaviours among adolescents and youth with T2DM. A meta-synthesis of qualitative studies was performed. Systematic search using PubMed, Scopus, Web of Science and CINHAL was conducted to identify relevant qualitative studies. Articles that fulfilled inclusion and exclusion criteria were iteratively read and independently coded by three investigators. Thematic analysis approach was used. All data were managed using NVivo 12. Nine qualitative studies were included in the meta-synthesis. All studies were conducted in the developed Western countries. A total of three sub-categories (i.e. knowledge, health status and co-morbidities, and skills) collectively form the overarching theme of intrapersonal factors influencing diabetes self-management among adolescents and youth with T2DM. Our findings suggested that the patients equipped with a good disease knowledge understand the importance of self-management, and this helps them to adhere with suggested diabetes self-care activities. Poor health status and co-morbidities were perceived as barriers as the adolescent felt that these added complexity to self-management and interfered the treatment goal. In addition, adolescents perceived planning and coping skills essential in overcoming challenges that may interrupt their diabetes self-management. The current study elicited having good knowledge, planning and coping skills as facilitators in which lack of these factors pose challenges in diabetes self-management. The young patients should be empowered with necessary knowledge and skills to sustain daily diabetes self-management, with particular attention to those with co-morbidities and poor health status. As all articles included in this meta-synthesis were from Western countries, more studies from different settings and countries are crucial in order to design interventions which suit the need of adolescents and youth with T2DM in disease self-management.

## 44 Outcome of Prescription Filling Service by a Community Pharmacy in Thailand

### Sunee Lertsinudom^1,3^, Pawalee Niamtaworn^2^, Sirirat Tanpichart^3^

#### ^1^Division of Clinical Pharmacy, Faculty of Pharmaceutical Sciences, Khon Kaen University; ^2^Division of Clinical Pharmacy, Faculty of Pharmacy, Thammasat University; ^3^Community Pharmacy Association (Thailand)

In Thailand, department of pharmacy in each hospital is responsible to fill and dispense all medications for both in- and out-patients. The workload of the department is overwhelming and causing with long-waiting time for using the hospital service. Due to this inconvenience, the community pharmacy association (Thailand) has built up a model called Prescription Filling Service by community pharmacy and launched the program in a province of Northeastern, Thailand. This program is co-working with a local hospital to improve the prescription filling time for these patients. This research was aims to evaluate the program in terms of management of medication-related problems by community pharmacist and satisfaction and opinion of both service provider and patients. Factors affecting patient’s willingness to pay for the program and factors affecting pharmacist to provide this service were being defined. Data were collected retrospectively from the service’s database about medication-related problems management by community pharmacists. Semi-structured interview was used to assess satisfaction and opinion from both patients and pharmacists participating in the program from June 2017 to November 2018. There are 27 community pharmacists and 137 patients participated in the service. The pharmacists managed 17 problems (problems solving for 13 cases, problems prevention for 3 case and advisement for 1 case). From interviewing 66 patients and 18 pharmacists were extremely satisfied and very satisfied for the service, respectively. The patients’ waiting time for receive medicines at community pharmacy (average 1.14 min) was significantly less than when waiting at the hospital (average 67.79 min), p<0.001; likewise traveling period, traveling expenses and opportunity cost for working. 82.54% of patients were willing to pay for the service and factors affecting patient’s willingness to pay were gender, age and level of education (Fisher's Exact Test; P = 0.008, 0.043 and 0.048, respectively). 94.44% of pharmacists will continue the service even there is no financial support from sponsor. Gender, age, type of community pharmacy and community pharmacy relations did not affect service provided by pharmacists (Fisher's Exact Test; P > 0.05). Prescription filling service by community pharmacy is useful for outpatients as it reduces waiting time at hospital. Community pharmacy is the valuable resource that could help in managing drug-related problems along with providing time convenience for these patients. Therefore, this prototype program should be supported and developed throughout the country

## 45 General Public Views Regarding the ADRs Reporting System in Dubai-UAE

### Doaa Alkhalidi^1,2^, Shazia Qasim Jamshed^2^, Ramadan Mohamed Elkalmi^3^, Mirza Rafi Baig^4^, Adeel Aslam^2^ Mohamed Azmi Hassali^5^

#### ^1^ Department of Clinical Pharmacy & Pharmacotherapeutics, Dubai Pharmacy College, Dubai, UAE; ^2^ Department of Pharmacy Practice, Kulliyyah of Pharmacy, IIUM, Kuantan, Pahang 25200, Malaysia; ^3^ Department of Pharmacy Practice, Faculty of Pharmacy, Universiti Teknologi Mara, Shah Alam; ^4^ Department of Clinical Pharmacy & Pharmacotherapeutics, Dubai Pharmacy College, Dubai, UAE; ^5^ Discipline of Social and Administrative Pharmacy, School of Pharmaceutical Sciences, Universiti Sains Malaysia, Penang 11800, Malaysia.

##### **Correspondence:** Shazia Qasim Jamshed (shazia_jamshed@iium.edu.my)

Active participation of consumers toward adverse drug reactions (ADRs) reporting system is vital to enhance medication safety. The current research is aimed to determine the public awareness and views regarding ADRs reporting in UAE. This cross-sectional was executed in different public areas of Dubai from February to May 2018. A self-administered questionnaire was developed, validated and piloted. A convenient sample of 400 individuals from general public was recruited. The collected questionnaires were treated anonymously and analyzed statistically. Only 15.8% of participants documented their awareness towards the existence of ADR reporting system in UAE. More than 55% encouraged labeling the information (of the established ADR reporting system) on the package of the medicines, while (37%) suggested establishing awareness campaigns in the community and (28%) recommended pharmacist’s contribution in this regard. Half of the respondents (50%) preferred to report ADRs by speaking face to face to qualified personnel while 32% preferred reporting through telephone and 20% by filling online forms. More than 90% agreed that reporting of ADRs is beneficial to public and this, therefore, strengthen drug safety and prevent recurrence of adverse drug reactions. The established ADR reporting system was not known to most of the participants. However, public have shown high interest in reporting ADR in near future. Robust interventions are required to increase public awareness about the importance of their contribution towards national pharmacovigilance system in UAE.

## 46 Treatment Factors Affecting Medication Adherence among Elderly Living in Long-Term Care Facilities

### Ezlina Usir, Nurul Hafizah Azman, Hasnah Ismail, Mathumalar Loganathan Fahrni

#### Department of Pharmacy Practice, Faculty of Pharmacy, Universiti Teknologi MARA (UiTM) Cawangan Selangor, Kampus Puncak Alam, 42300, Selangor, Malaysia.

##### **Correspondence:** Ezlina Usir (ezlin365@uitm.edu.my)

Long-term care (LTC) facilities provide a long-term care among elderly population. Most elderly are at risk of needing this long-term care due to older age and physical disability. Several studies have indicated that LTC facilities residents are prescribed with more medications compared to community-dwelling elderly. This issue increases the risk of medication non-adherence which may lead to higher cost in healthcare and mortality rate among this population. Hence, this study aimed to assess risk of medication non-adherence and to identify treatment factors that affect medication adherence among elderly living in LTC facilities. One-to-one interview was conducted with 258 elderly living in LTC facilities in Selangor and Kuala Lumpur using a structured questionnaire. Elderly aged 65 or more, took at least one medication and have stayed at least one year in the LTC facilities were included in this study. Descriptive statistics and Pearson correlation test were used to analyse the findings. This study was approved by UiTM Research Ethics Committee, approval reference REC/242/19.Majority of residents were at low risk of medication non-adherence. A weak positive correlation was found between higher risk of medication non-adherence with difficulty in taking many tablets at the same time (p= 0.009) and in opening or closing the medication bottle (p= 0.040); increase occurrence of stopping medication when felt better (p=0.001) and felt worse (p= 0.028), changing dose to suit preference (p= 0.008) and feeling hassled to stick with dose schedule (p= 0.008). Behaviour of respondents towards medication taking, regimen complexity and difficulty to open and close the medicine bottles influence medication adherence among elderly living in LTC facilities. Hence, it is important for care providers to have an excellent professional relationship with this population in ensuring medication adherence.

## 47 Resource use and direct medical cost associated with different states of breast cancer in Malaysia

### Noor Razilah Abdul Rafar, Chin Fen Neoh, Muhamad Faiz Othman

#### Faculty of Pharmacy, Universiti Teknologi MARA, Puncak Alam, Selangor, Malaysia

Breast cancer is the most common type of cancer among females in Asia-Pacific region including Malaysia. However, little is known about the resource use and direct medical cost associated with different states of breast cancer. Hence, this study aimed to identify the type of resource use and to determine the direct medical cost associated with different states of breast cancer in Malaysia. This study retrospectively retrieved data to estimate the direct medical cost associated with the different states of breast cancer patients recruited from two main oncology centres [i.e. Hospital Sultan Ismail and Institut Kanser Negara (IKN)]. Patient were categorized into four states (P, R, S, M). P state had a primary diagnosis breast of cancer within 1 year from the point of data collection and no metastatic disease. Patients in state R had at least one recurrence within 1 year and no metastatic disease while patients in S state were in the second and following years after primary breast cancer. Patients with at least one distant recurrence were categorized as M state. Direct medical costs included the costs incur at both outpatient and inpatient settings. All cost data were reported in 2019 Malaysian Ringgit and all data were then analysed using SPSS version 25. A total of 383 patients were included in this study. The mean age was 51.2 ± 10.3 years old and most were Malay (n=252, 65.8%). The majority of the patients were in P state (n=134, 35.2%), followed by S state (n=129, 33.7%), M state (n=76, 19.8%) and R state (n=44, 11.5%). Patients in the P state had the highest total direct mean cost amounted to RM25,086.36 per patient, followed by R state (RM19,218.98), M state (RM16,917.96) and S state (RM3,761.92). This could be due to the higher treatment cost incurred during the first year of primary breast cancer. In the Malaysian setting, the total direct medical cost was substantially higher when compared to the following years.

## 48 Quality of Life Perceptions’ among Government Servants

### Nur Zuhaida Zainal Bahrin^1^, Wan Sazrina Wan Zaid ^2^, Fauziah Zamri^3^

#### ^1,^ Klinik Kesihatan Senawang, Ministry of Health, Malaysia ^2^ Faculty of Pharmacy, Cyberjaya University College of Medical Sciences Malaysia

##### **Correspondence:** Nur Zuhaida Zainal Bahrin (zuhaidazb@gmail.com)

Quality of life (QoL) is one of the crucial factors that will influence individual performance in their life. The perception on quality of life has become one of the major issues discussed globally. Ministries in public service in Malaysia have set up a vision for the nation to work together for better health which includes ensuring good quality of life among their staff. Therefore, this study was done to know the perception on QoL and factors affecting it. A quantitative cross-sectional study was conducted in Selangor and Putrajaya which involved government servants from health and education division. Questionnaire consisted of two major sections which were demographic data and six QoL components was distributed to a total of 311 respondents. Respondents were recruited through convenient sampling. The data was collected from the respondents and later were analysed using Statistical Package for Social Sciences (SPSS) version 20 and data was presented using frequency tables and graphs. This study revealed that respondents perceived QoL on most of the components except for component of life enjoyment. The mean value for component of overall quality of life showed significant difference between male and female (p= 0.03). There was a negative correlation between age and quality of life components of life enjoyment (p= 0.004, r= -0.163). In addition, the mean values for physical state, life enjoyment and overall quality of life showed significant difference between the groups of marital status (p= 0.023, p<0.05, p=0.011). The responsibilities and challenges endured by both genders made the perception scores on quality of life significantly differs. The life commitments and responsibilities shared by elderly might be the underlying reason for their lower scores obtained compared to the younger group of age respondents. The marital status of our respondents reflected their perception on their physical state, life enjoyment and overall quality of life assessment. The outcome of married couples had higher mean values for certain components might be attributed to the fact that married couples had gained experience in managing their emotions better and compromised on the standards of happiness for overall impression on QoL. The perception on QoL is an important aspect in ones’ life as their perception might influence the way they lead their routine life. This study has shown that gender, age, and marital status are among the factors that affect the perception on QoL significantly.

## 49 The Comparisons of Treatment Cost Sciatica Patient Between NHI Members and Non-NHI Members

### Nurita Andayani, Farida Ariani, Prih Sarnianto

#### Faculty of Pharmacy, Pancasila University, Jakarta, Indonesia

##### **Correspondence:** Nurita Andayani (nurita.andayani@univpancasila.ac.id)

National health insurance has been held in Indonesia in 2014. One of the diseases guaranteed by National Health Insurance (NHI) is Sciatica. Sciatica, if left over, will lead to weakness of the lower limbs / lower limbs, accompanied by a decrease in the muscles of the lower limbs. These conditions can reduce productivity for sufferers. One treatment in Sciatica patients is by pharmacological means that is by administering drugs and non-pharmacological namely by means of physiotherapy. Hospital as a service provider provides two types of services both with NHI and without NHI for patients. The study aimed to know the difference in costs incurred in sciatica patients in their treatment both to NHI members Non-NHI members. The results of the study can be used as recommendations whether the therapy given to patients of NHI members is as effective as patients Non-NHI members. The study used 256 patients of dr. Suyoto Hospital, Jakarta, Indonesia that divided into two groups; they are 128 patients of NHI members and 128 patients of Non-NHI members. The aata was obtained by retrospectively and the study used cross sectional design. Data analysis in this study used t-test for two independent samples. The results showed that the costs incurred by sciatica patients with NHI members were significantly different from patients Non-NHI members (p-values = 0.000; p-value <5%). The mean cost of sciatica patients Non-NHI member was lower than patients NHI member (p-value= 0.000) but the variability of sciatica patient Non-NHI member is higher than variability for NHI member. This condition caused sciatica patients Non-NHI member have fewer hospital visits and greater pain relief than sciatica patients NHI members.

## 50 Knowledge, Attitude and Quality of Life of Patients with Type 2 Diabetes Mellitus in Malaysia

### Zarith Sofia^1^, Faiza Naimat^1,2^, Mathumalar Loganathan Fahrni^2^

#### ^1^School of Pharmacy, Management & Science University, 40100, Shah Alam Malaysia; ^2^Universiti Teknologi MARA Cawangan Selangor Kampus Puncak Alam

##### **Correspondence:** Faiza Naimat (naimatfaiza@gmail.com)

Type 2 diabetes mellitus (T2DM) is increasingly a major health concern in Malaysia. In the recent 2015 National Health and Morbidity Survey (NHMS, 2015), it was reported that prevalence of T2DM in Malaysia is 15.2% for adults aged 18 years and above. This figure is expected to double to 30% in the next 15 years. It has been postulated that knowledge of patients about their condition will affect their attitude and thereby their quality of life. Self-care in diabetes is vital for improving Health-related Quality of Life (HRQoL). This study aimed to evaluate the knowledge, attitude and HRQoL among T2DM patients. A cross-sectional study was conducted in the area of Shah Alam, Malaysia. The Michigan Diabetic Knowledge Test (MDKT), EuroQoL-five-dimentional (EQ-5D) and EuroQoL-visual analogue scale (EQ-VAS) questionnaires were used as instruments to assess patient knowledge, attitude and HRQoL. The sample consisted of predominatly females (58.6%), with 41.5% of having a range of age from 56-65. The mean age was 57 ± 13.3 and 40% were diagnosed with T2DM in the past 6-10 years. Among 111 respondents, 15.3% had poor knowledge; 71.2% had moderate knowledge and 13.5% had good knowledge. The mean score for knowledge was 8.95 ± 2.34 which showed moderate knowledge. The average attitude score of all respondents was 3.67 ± 3.1 which depicted a negative attitude. A moderate level of HRQoL (57.8 ± 20.9) was recorded in the study. There was a significant positive association between knowledge and attitude (p=0.001), knowledge and HRQoL (p=0.005) and in addition between attitude and HRQoL (p=0.025). Knowledge and QoL levels were moderate among patients with T2DM in Shah Alam, Malaysia. Improved knowledge translated into positive attitudes, which in turn translated into improved HRQoL.

## 51 Knowledge, Attitude, Current Practice and Barriers of Kuala Lumpur Community Pharmacists towards Mental Health Care

### Beh Huai Min^3^, Nor Liana Che Yaacob^1,2^, Irma Wati Ngadimon^3^, Mathumalar Loganathan Fahrni^2^

#### ^1^Unit of Pharmacy Practice, School of Pharmacy, Management and Science University, University Drive, Off Persiaran Olahraga, 40100 Shah Alam Selangor, Malaysia; ^2^ Department of Pharmacy Practice, Faculty of Pharmacy, Universiti Teknologi MARA Cawangan Selangor Kampus Puncak Alam, 42300 Bandar Puncak Alam, Selangor, Malaysia; ^3^Department of Pharmacy Practice, Faculty of Pharmacy, Mahsa University, Jln SP 2, Bandar Saujana Putra, 42610 Jenjarom, Selangor, Malaysia

##### **Correspondence:** Nor Liana Che Yaacob (nor_liana@msu.edu.my), (Lyanas76@yahoo.com)

Deterioration in mental health is an increasing concern worldwide. The prevalence of mental illness in Malaysia is increasing each year. Based on a study by the National Health and Morbidity Survey (NHMS) in 2015, 4.2 million Malaysians aged 16 years and above (29.2%) suffered from various mental health issues ranging from depression to suicide. Pharmacotherapy plays a role in treating mental health issues. Community pharmacists as one of the primary healthcare providers can play an integral role in optimising medication use in patients as well as managing side effects. However, little is known about the level of knowledge and attitudes of community pharmacists regarding mental health as well as to barriers to delivering effective counselling to patients with the mental illness. This cross-sectional study was conducted among community pharmacists in Klang Valley, Malaysia. The instrument used was a questionnaire adopted and adapted from 2 research papers; Watkins, et al. (2017) and Owusu-Daaku et al. (2010). Data was then analysed using Statistical Package for Social Science (SPSS) version 22. Data on demographics and dependent variables (eg. role, knowledge, perception and barrier) were presented with as means and percentages. Chi-square was used to determine association between demographic data with perception, confidence level, knowledge and interest levels. From the X number of questionnaires distributed, 236 community pharmacists returned with responses to the questionnaires. Pharmacists had limited knowledge on managing patients with mental health concerns. Nevertheless, they had positive attitudes and were willing to undergo continuous training programs to improve their knowledge regarding antipsychotics. Pharmacists perceived lack of knowledge, lack of privacy for patients and lack of time as main barriers to delivering effective counselling to patient with mental illnesses. Despite having limited knowledge, community pharmacists demonstrated positive attitudes and acknowledged the importance of providing effective counselling to patients. Training and education on effective mental health management can be of assistance.

## 52 Exploring the Effectiveness of Islamic Practices among MMT Clients in Klinik Cure&Care 1Malaysia Sg. Besi, Kuala Lumpur: A Qualitative Study

### Safwanah Rahman^1^, Nasrul Nazim Husna Zunaidi^1^, Azyyati Mohd Suhaimi^2^

#### ^1^Faculty of Pharmacy, Universiti Teknologi MARA (UiTM) Selangor, Kampus Puncak Aam, 42300 Bandar Puncak Alam, Selangor; ^2^Department of Pharmacy Practice, Faculty of Pharmacy, Universiti Teknologi MARA (UiTM) Selangor, Kampus Puncak Aam, 42300 Bandar Puncak Alam, Selangor.

Various strategies have been implemented in different countries to reduce and eradicate the supply and demand to substance abuse. Cure & Care 1Malaysia (CC1M) clinic was introduced by the Agensi Antidadah Kebangsaan (AADK) in 2010 following the government steps on transforming the treatment program for drug addicts in the country. The programme has adopted Islamic practices as one of their primary rehabilitation components, in addition to offering a pro-social environment to help their recovery. This study aimed to qualitatively decipher the effectiveness of Islamic practices applied in the program on clients receiving Methadone Maintenance Therapy (MMT) in CC1M. All male Muslim clients aged between 26-45 years old were selected and individually interviewed face-to-face for 20 minutes using a validated questionnaire to collect the data using an audio recorder. Each client was identified using a code to preserve their confidentiality. The audio files were transcribed line-by-line into Microsoft Word to get familiarization with the data and themes were extracted when statements are emphasized. The results revealed that all clients showed their preference in performing the daily or sunnah prayers and reciting al-Quran with strong compliances with the religious activities at the clinic. It was also observed that the recovery from drug abuse was aided by the application of Islamic practices in their lives, and thus should be continuously be embedded in their daily routine due to the positive outcomes.

## 53 Compliance of Natural Product Advertisements Published in Local Magazines toward Current Regulations in Malaysia

### Manan MM^1^, Jamaluddin NL^2^

#### ^1^Faculty of Pharmacy, UiTM, Puncak Alam Campus; ^2^School of Pharmacy, KPJ Healthcare University College, Nilai

Availability of natural products promoted through advertisement in the Malaysian market is remarkably high. Various platforms of advertising these products straight to the consumer and claims of products health benefit might be the reason for which the prevalence of natural product use is high among Malaysian. The products claim may not be necessarily true and can affect consumers’ knowledge and perception towards the products and the safety of the consumers is now being questioned. Therefore, this study aims to examine the compliance of these product advertisements in magazines towards current regulations and guidelines in Malaysia. An observational study was conducted to screen natural product advertisements published in local magazines and then compared with the criteria outlined by Malaysian Advertisement Board regulations and guidelines 2017. Only magazines published within the periods of January 2016 to December 2017 were selected and screened. The magazines selected were *Mingguan Wanita*, *Health Today*, *Laman Impian and Glam Lelaki*. A total of 450 advertisements from 144 different natural products were reviewed. Natural products were found most advertised in *Mingguan Wanita* magazines where it dominates 87.8% of the advertisement. Most of the products (n = 73, 50.7%) are unregistered and few of the product advertisements contained exaggerating claims. The findings showed that some of the natural product advertisements published in local magazines are not complying with the current regulations in Malaysia. Unregistered products and exaggerated claims can posed a possible threat to consumer safety risk since the exposures can lead to possible unwanted side effects or toxicity. Furthermore, these findings can be used by the National Pharmaceutical Regulatory Agency to conduct a more comprehensive pharmacovigilance activities towards non-compliant advertisements of natural products. This effort will eventually prevent the potential harms to the consumers.

## 54 Compliance of Cosmetic Products Advertisements with Malaysian Regulation

### Manan MM^1^, Nadiah Loke AL^2^

#### ^1^Faculty of Pharmacy, UiTM, Puncak Alam Campus; ^2^School of Pharmacy, KPJ Healthcare University College, Nilai

The status of compliance of cosmetic product advertisements is dependent on the pharmacovigilance and enforcement by the National Pharmaceutical Regulatory Agency in Malaysia. The control of cosmetic can avoid the consumer from receiving a false and exaggerated information which can be a threat to the consumer safety. Due to lack of study on this, this research was conducted to evaluate the compliance of the advertisements with the cosmetic advertising code guidelines provided by the Ministry of Health Malaysia. This is a 3 months descriptive study with no intervention. The data was collected, analysed and evaluated randomly 2 Malay language magazine and 2 English language magazines in Malaysia. A total of 660 advertisements were screened and of these 202 were from international companies. On comparison between local and international companies advertisements, non-compliant were mostly from local companies. The findings of this study showed not all companies comply with the current guidelines and regulations. There is a need to monitor cosmetic advertisements with regards to compliance to the current guidelines and regulations.

## 55 A Study on the Perception and Knowledge Related to Herbal Medicine among UiTM Students

### Muhammad Anwar Nawab Khan

#### Department of Pharmacy practice, Faculty of pharmacy, Universiti Teknologi MARA (UiTM), 42300 Bandar Puncak Alam, Selangor, Malaysia

The purpose of this study is to find out what is the perception and level of knowledge towards herbal medicine among pharmacy students in UiTM Puncak Alam. A cross sectional study by using stratified sampling was disseminated among 264 pharmacy students with different year of study. Data were collected using a validated questionnaire for each respondent that consist of of 4 sections (Demographic background, General Experience, Perception and Knowledge). This study revealed that most of pharmacy students have positive perception about herbal medicines. The fourth year students proved that they have higher level of knowledge compared to others. This study also showed that there was significant association between knowledge and different year of study. Third year and fourth year students succeeded to get the high score for level of knowledge while first and second year students seem to have inadequate knowledge. Majority of pharmacy students had positive perception towards herbal medicine regardless the current year of study. Actions should be taken to ensure pharmacy students are aware about the safety of herbal medicines.

## 56 Knowledge, Awareness and Perception of Tuberculosis among Residence in Puncak Alam and Kerteh

### Nurin Alya Bt Saroge, Muhammad Anwar Nawab Khan

#### Department of Pharmacy practice, Faculty of pharmacy, Universiti Teknologi MARA (UiTM), 42300 Bandar Puncak Alam, Selangor, Malaysia

Tuberculosis (TB) is an infectious disease which is transmitted through by air. The disease is highly contagious and easily spreads when infected people cough, sneeze, spit or talk. Unfortunately, tuberculosis usually mistaken as common cough as the symptoms are quite similar to each other. This lack of knowledge and awareness is a problem which can lead to increment of occurrence of the disease. Therefore, the study attempts to examine the level of knowledge, awareness and perception of tuberculosis among residence in Puncak Alam and Kerteh. This research used cross sectional study and convenience sampling which included sample size of 400 with 200 from Puncak Alam and 200 from Kerteh. Disseminated questionnaire was used as a method of study and the results were analysed using IBM SPSS statistic 25.0. The findings indicated that the respondents have moderate knowledge but good awareness level regarding the disease. It was also found that the disease was negatively perceived by the respondents. However, most of the respondents also show positive response towards infected people.

## 57 A Retrospective Cross-sectional Review of Adverse Drug Reactions Reported in Sultanah Aminah Hospital Johor Bahru

### Wang S. Ng^1^, Pey C. Wong^1^, Siti N. Md Said^1,2^

#### ^1^Department of Pharmacy, Sultanah Aminah Hospital, Johor Bahru, Malaysia; ^2^Faculty of Pharmacy, Universiti Institute technology MARA, Puncak Alam, Malaysia

Adverse drug reaction (ADR) often leads to hospital admission, prolongation of hospital stay and emergency department visits that result in clinical burden and economic costs. Early identifying and preventing of ADRs in every patient is crucial so as to ensure the safe patient care. There is little information about hospital-based studies on ADRs in Malaysia. The aim of the study was to study the pattern of ADRs reported in Sultanah Aminah Hospital Johor Bahru (HSAJB). All ADRs reported by healthcare providers to the Drug Information Services HSAJB IN January 2014 to December 2015 were collected. Report forms with incomplete data were excluded. Suspected medications contributing to an ADRs, the pharmacological groups, ADR categories, extent of reaction and causality assessment were collected among others. Descriptive analysis was performed using Microsoft Excel. Total numbers of 295 drugs were reported. Anti-infectives (35.3%), drugs affecting the central nervous system (22%), and cardiovascular (CVS) (12.2%) were the three main pharmacological groups involved. Extent of reaction varies for each drug; cutaneous (54.2%), CVS (10.8%) and haematologic reactions (7.1%) the most reported. The five main drugs with the ADRs were allopurinol (6.4%), phenytoin (6.4%), warfarin (6.1%), bactrim (6.1%) and nevirapine (6.1%). Allopurinol and phenytoin mostly lead to severe ADRs, with 13(4.4%) and 10(3.4%) cases respectively. In conclusion, it is important to identify the main drugs that are involved in ADRs so that healthcare professionals are aware while prescribing these medicines to patients.

